# Assessment of the real-time PCR and different digital PCR platforms for DNA quantification

**DOI:** 10.1007/s00216-015-9107-2

**Published:** 2015-10-31

**Authors:** Jernej Pavšič, Jana Žel, Mojca Milavec

**Affiliations:** Department of Biotechnology and Systems Biology, National Institute of Biology, Večna pot 111, 1000 Ljubljana, Slovenia; Jožef Stefan International Postgraduate School, Jamova 39, 1000 Ljubljana, Slovenia

**Keywords:** Digital PCR, Real-time PCR, Molecular diagnostics, Human cytomegalovirus, DNA quantification

## Abstract

Digital PCR (dPCR) is beginning to supersede real-time PCR (qPCR) for quantification of nucleic acids in many different applications. Several analytical properties of the two most commonly used dPCR platforms, namely the QX100 system (Bio-Rad) and the 12.765 array of the Biomark system (Fluidigm), have already been evaluated and compared with those of qPCR. However, to the best of our knowledge, direct comparison between the three of these platforms using the same DNA material has not been done, and the 37 K array on the Biomark system has also not been evaluated in terms of linearity, analytical sensitivity and limit of quantification. Here, a first assessment of qPCR, the QX100 system and both arrays of the Biomark system was performed with plasmid and genomic DNA from human cytomegalovirus. With use of PCR components that alter the efficiency of qPCR, each dPCR platform demonstrated consistent copy-number estimations, which indicates the high resilience of dPCR. Two approaches, one considering the total reaction volume and the other considering the effective reaction size, were used to assess linearity, analytical sensitivity and variability. When the total reaction volume was considered, the best performance was observed with qPCR, followed by the QX100 system and the Biomark system. In contrast, when the effective reaction size was considered, all three platforms showed almost equal limits of detection and variability. Although dPCR might not always be more appropriate than qPCR for quantification of low copy numbers, dPCR is a suitable method for robust and reproducible quantification of viral DNA, and a promising technology for the higher-order reference measurement method.

**Electronic supplementary material:**

The online version of this article (doi:10.1007/s00216-015-9107-2) contains supplementary material, which is available to authorized users.

## Introduction

Real-time PCR (qPCR) is a PCR-based method for quantification of nucleic acids. Owing to its dominant performance over other quantification methods in terms of accuracy, specificity, repeatability and dynamic range, qPCR is routinely used for various applications in agriculture, medicine, molecular diagnostics, forensic testing and testing of genetically modified organisms [1]. In recent years, a novel version of PCR, known as digital PCR (dPCR), has become widely used in the field of nucleic acid quantification. Digital PCR (dPCR) uses limiting dilutions and sample partitioning into sub-microlitre reactions to achieve sensitive, precise, accurate, reliable and reproducible quantification of nucleic acids. In contrast to analogue qPCR, where the amplification signal is logarithmic and the quantification is based on external calibration, dPCR offers simple, linear and digital quantification that is based only on the number of positive and negative reactions, with the Poisson distribution taken into account [2, 3]. Digital PCR (dPCR) has additional advantages over qPCR, as it has been reported to be more tolerant to some PCR inhibitors, sequence variations and different types of DNA templates, PCR assays and master mixes [2, 4–6]. Although there are some limitations of dPCR owing to the restricted reaction volume, which can result in lower analytical sensitivity compared with qPCR [7–9], dPCR offers high repeatability even when measuring low DNA concentrations [2, 9, 10]. Because of these benefits of dPCR, it has great potential and has already been used for many different applications, such as for plant pathogens and testing of genetically modified organisms, detection of resistant bacteria, viral diagnostics, rare-mutant detection and copy-number variations [11–16].

This digital format (i.e., dPCR) is achieved either by a microfluidic-based approach, where a reaction is divided into hundreds or thousands of chambers on a single plate or array, or by a droplet-based approach, where a reaction is separated into thousands or millions of droplets [8]. Despite the availability of several different dPCR platforms, most studies have been performed either with the microfluidic-based Biomark™ HD system (Fluidigm) [2, 6, 17] or with the droplet-based QX100™ Droplet Digital™ PCR system (Bio-Rad) and the QX200™ Droplet Digital™ PCR system (Bio-Rad) [18–20]. Although many analytical characteristics of the QX100 system and the 12.765 Digital Array™ integrated fluidic circuit (Fluidigm) on the Biomark system have already been determined and compared with those of qPCR [2, 5, 9], to the best of our knowledge, no assessment of the qdPCR 37 K™ integrated fluidic circuit (Fluidigm) on the Biomark system in terms of linearity, analytical sensitivity and limit of quantification (LOQ) has been performed, although it offers high-throughput analysis. Additionally, only a few studies have focused on systematic direct comparisons between qPCR and both of these dPCR platforms using the same DNA material. Also, for each dPCR platform, the influence of different PCR components on DNA quantification has been poorly studied.

**Fig. 1:**
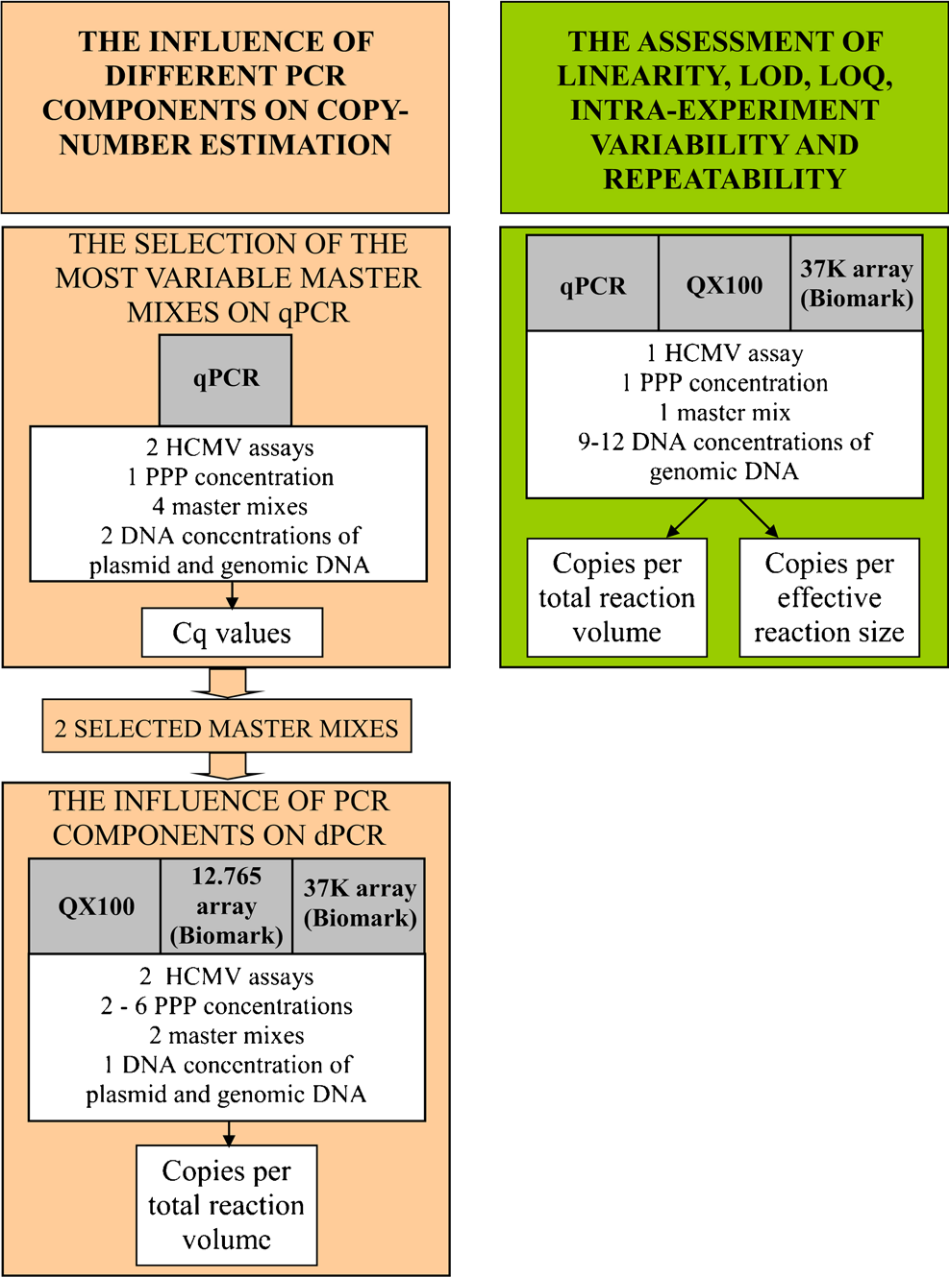
The experimental procedures. *Cq* quantification cycle, *dPCR* digital PCR, *HCMV* human cytomegalovirus, *LOD* limit of detection, *LOQ* limit of quantification, *PPP* forward primer, reverse primer and probe, *qPCR* real-time PCR

Here, qPCR and the two dPCR platforms, the QX100 system and the Biomark system (using the 12.765 array and the 37 K array), were assessed with use of clinically relevant concentrations of two reference materials for human cytomegalovirus (HCMV), one of which contained circular plasmid DNA, and the other contained linear genomic DNA (gDNA) (Fig. 1). For the QX100 system and for both arrays of the Biomark system, the effects of different PCR components [HCMV assay; forward primer, reverse primer and probe (PPP) concentrations; master mixes] on the final quantification of each DNA template type was analysed. Additionally, qPCR, the QX100 system and the 37 K array were evaluated in terms of linearity, limit of detection (LOD), LOQ and intraexperiment and interexperiment variability (repeatability). Finally, to gain further knowledge of the analytical performances of each of these dPCR platforms, two different approaches were used, one considering total reaction volume and the other considering effective reaction size.

## Materials and methods

### Reference materials

#### Plasmid DNA

Standard Reference Material 2366 Cytomegalovirus (CMV) for DNA Measurements, composed of HCMV Towne_∆147_ bacterial artificial chromosome DNA, was purchased from the National Institute of Standards and Technology (USA) [21]. Initially, 150 μL component C (declared concentration, 19,641 copies per microlitre) was diluted in 1.35 mL 1× tris(hydroxymethyl)aminomethane–EDTA buffer (Sigma-Aldrich, USA), and aliquoted and stored at −80 °C [7]. As the National Institute of Standards and Technology material is composed of purified plasmid DNA, no further DNA extraction was needed before its amplification. Because of plasmid instability reports from the manufacturer, aliquots were tested for the concentration and stability on the QX100 system by means of the *UL54* assay (*UL54* is a DNA polymerase gene of HCMV). The mean aliquot concentration (± standard error) was estimated as 1050 (±30) copies per microlitre (henceforth indicated as the nominal concentration).

#### Genomic DNA

The 1st WHO International Standard for Human Cytomegalovirus for Nucleic Acid Amplification Techniques was purchased from the National Institute for Biological Standards and Control (code 09/162) [22]. This comprised a lyophilized whole-virus preparation of the HCMV ‘Merlin’ strain. After reconstitution in 1 mL double-distilled water, the material was additionally diluted fivefold in phosphate-buffered saline (137 mM NaCl, 2.7 mM KCl, 8 mM Na_2_HPO_4_, 2 mM KH_2_PO_4_, pH 7.4). Twenty-two 200-μL aliquots were prepared and extracted on the same day, using High Pure Viral Nucleic Acid kits (Roche), according to the manufacturer’s instructions. The extracted gDNA was then pooled, mixed, aliquoted and stored at −20 °C. The concentration and stability of the gDNA were evaluated on the QX100 system by means of the *UL54* assay. The mean nominal concentration was 2580 (±60) copies per microlitre.

### Amplification methods

#### Real-time PCR

The qPCR was performed on a Viia™ 7 Real–Time PCR System (Life Technologies, USA) according to the Minimum Information for Publication of Quantitative Real-Time PCR Experiments (MIQE) guidelines [1] (Table S1). For the selection of the most variable master mixes, 10-μL reaction mixtures comprising 3.5 μL DNA sample, 0.5 μL HCMV assay (the *UL54* assay or the *UL83* assay; *UL83* is the phosphoprotein pp65 gene of HCMV), and either 3.5 μL double-distilled water and 2.5 μL 4× master mix (TaqMan® Fast Virus 1-Step Master Mix; Applied Biosystems, USA) or 1 μL double-distilled water and 5 μL 2× master mix [TaqMan® Universal PCR Master Mix, Applied Biosystems, USA; or TaqMan® Gene Expression Master Mix, Applied Biosystems, USA; or FastStart Universal Probe Master (Rox), Roche Diagnostics, Switzerland]. Henceforth, each of these four master mixes are abbreviated, respectively, as ‘Fast master mix’, ‘Universal master mix’, ‘Gene master mix’, and ‘Roche master mix’. The assessment of the linearity, LOD, LOQ, and intraexperiment variability and repeatability was performed with 20-μL reaction mixtures of 8 μL DNA sample, 1 μL HCMV assay, 6 μL double-distilled water, and 5 μL 4× Fast master mix. For amplification, MicroAmp® EnduraPlate™ Optical 384-Well Clear Reaction Plates with Barcode (Life Technologies) were used. The reactions were conducted under the universal conditions of 2 min at 50 °C, 10 min at 95 °C, and 45 cycles of 15 s at 95 °C and 1 min at 60 °C. Analysis of the data was performed with Viia™ 7 Software version 1.2.4. For each master mix, one negative template control (NTC) was used.

#### Droplet-based dPCR

For the QX100 system, all of the reactions were performed according to the digital MIQE guidelines [23] (Table S2). The 20-μL reaction mixtures consisted of 8 μL sample, 1 μL HCMV assay, 1 μL double-distilled water, and 10 μL 2× ddPCR™ Supermix for Probes (Bio-Rad Laboratories, USA). For each combination of PCR components, the relevant NTCs were included. A QX100™ droplet generator (Bio-Rad) was used to generate the droplets. The reactions were performed on the GeneAmp® PCR System 9700 (ABI) under the same universal conditions as for the qPCR, with the addition of 10 min at 98 °C. After amplification, the plate was loaded onto the QX100 system, where analysis of the droplet fluorescence was done with QuantaSoft Software version 1.3.2.0 (Bio-Rad). The fluorescence was monitored over the FAM and HEX spectral regions. All of the thresholds were set up manually to allow the distinction between positive and negative droplets, and the concentrations of DNA were calculated with Eqs 1, 2, 3 and 4, given later. Only the reactions with more than 10,000 accepted droplets were used for analysis. As no positive droplets were observed in eight NTCs, a single positive droplet was enough to determine a sample as positive.

#### Microfluidic-based dPCR

For the Biomark system, all of the reactions were performed according to the digital MIQE guidelines [23] (Table S2). Two different arrays were used. To test the effects of different PCR components, the 12.765 array was used with 8-μL reaction mixtures comprising 3.2 μL sample, 0.4 μL HCMV assay, 0.5 μL 20× GE sample loading reagent (Fluidigm Europe, The Netherlands) and either 2 μL double-distilled water and 2 μL 4× Fast master mix, or 4 μL 2× master mix (as Universal master mix, Gene master mix or Roche master mix). To evaluate the influence of different PCR components on the 37 K array, the 4-μL reaction mixtures used comprised 1.4 μL sample, 0.2 μL HCMV assay, 0.4 μL 20× GE sample loading reagent, and either 1 μL 4× Fast master mix and 1 μL double-distilled water, or 2 μL 2× master mix (as Universal master mix, Gene master mix or Roche master mix). To assess the linearity, LOD, LOQ, and intraexperiment variability and repeatability on the 37 K array, the 4-μL reaction mixtures comprised 2.4 μL sample, 0.2 μL HCMV assay, 0.4 μL 20× GE sample loading reagent and 2 μL 4× Fast master mix. The loaded arrays were then transferred to the Biomark system. The reactions were performed under the same universal conditions as for the qPCR. The fluorescence was monitored over the FAM and HEX spectral regions. Data analysis was done with Biomark™ HD Data Collection Software version 3.1.4, with manual determination of the fluorescence threshold, the quality threshold (0.2) and the accepted quantification cycle (Cq) range (15–45 Cq). As no chambers with amplification were observed in six NTCs, one single positive chamber was enough to determine a sample as positive.

### Data processing for dPCR

Several equations were used to calculate the DNA concentrations measured with both of the dPCR platforms, as follows.

The mean number of copies per partition ($\lambda$) was calculated according to Eq. 1 [23]:

1 $$ \lambda =- In\left(1-\frac{k}{n}\right) $$

where *k* is the number of positive partitions, and *n* is the number of partitions analysed.

The total reaction volume is the complete volume of the reaction mix that is transferred into the inlet or the well. The number of estimated copies per total reaction volume (*M*) was calculated according to Eq. 2:

2 $$ M=\lambda \frac{total\kern0.5em  reaction\kern0.5em  volume\left(\mu L\right)}{partition\kern0.5em  volume\kern0.28em \left(\mu L\right)} $$

The effective reaction size is a certain portion of the total reaction volume that is transferred into the partitions analysed (chambers or analysed droplets), followed by use of the analysis software, whereas the rest of the total reaction volume was excluded from the analysis. The effective reaction size is the product of the number of the partitions analysed and their volume [23]. The number of estimated copies per mean effective reaction size (*N*) was calculated according to Eq. 3 [23]:

3 $$ N=\lambda n $$

The number of estimated copies per 1 μL sample (*C*) was calculated according to Eq. 4:

4 $$ C=d\frac{M}{sample\kern0.5em  input\kern0.5em  volume\;\left(\mu L\right)}, $$

where *d* is the dilution of the sample before the analysis.

### Determination of the total reaction volume and the effective reaction size

For each platform, the data for LOD, LOQ, and intraexperiment variability and repeatability were determined in two ways, according to either the total reaction volume or the effective reaction size (Eqs. 1, 2, and 3). For the qPCR, the total reaction volume was 20 μL, for the 37 K array for the Biomark system, it was 4 μL, and for the QX100 system, it was 20 μL. On the qPCR platform, in each well the complete 20-μL reaction was analysed for the fluorescence intensity in real time; therefore, the effective reaction size is similar to the total reaction volume. However, to determine the effective reaction size on the digital platforms, the number of partitions analysed and their volume need to be defined. For the 37 K array for the Biomark system, the number of partitions and their volume were already defined, as each panel contains 770 chambers of 0.84 nL; thus, the effective reaction size was 0.647 μL. For the QX100 system, the number of partitions analysed varied between each analysed inlet, and thus it needs to be determined individually with use of the information from QuantaSoft Software. In the present study, the number of accepted droplets ranged from 10,300 to 14,700, with a mean of 13,600. On the basis of their volume of 0.834 nL [18], the mean effective reaction size was estimated as 11.3 (±0.8) μL.

### Selection of the most variable master mixes on the qPCR platform

The qPCR study was initially done to select (1) one master mix that gave the highest variability for the Cq between the two HCMV assays; and (2) two master mixes that gave the highest difference in Cq values for the same HCMV assay. To achieve high variability, we examined two commonly used HCMV assays that target different genes and differ in amplicon size by more than twofold (Table S3). Both the *UL54* assay, which targets a DNA polymerase gene with the FAM/BHQ hydrolysis probe [24] (Table S3), and the *UL83* assay, which targets the phosphoprotein pp65 gene with the HEX/BHQ hydrolysis probe [25] (Table S3), were used in combination with each of the four master mixes (i.e., Fast master mix, Universal master mix, Gene master mix and Roche master mix). Both of these HCMV assays were used with 600 nM primers and 100 nM probes. To determine the PCR efficiency, each combination of HCMV assay and master mix was tested in duplicate, with either 1× diluted plasmid DNA and 5× diluted plasmid DNA, or 2.5× diluted gDNA and 12.5× diluted gDNA. A single NTC was performed for each combination of HCMV assay and master mix.

### Influence of different PCR components on DNA quantification by dPCR

For both of the dPCR platforms, each DNA template type was used and different PPP concentrations were tested with each HCMV assay (i.e., *UL54* and *UL83*). For the Biomark system, the Fast master mix and Universal master mix were used for the analysis, whereas for the QX100 system, the supermix for probes was used, as the only suitable master mix that allowed droplet stabilization. For the 12.765 array for the Biomark system, three different PPP concentrations (i.e., 300/300/100 nM, 600/600/200 nM, and 900/900/200 nM) were used with each combination of DNA template type, HCMV assay and master mix. For the 37 K array for the Biomark system, only the lowest two of these PPP concentrations were used. For the 12.765 array, the mixtures for duplicate reactions were composed of either 4.5× diluted plasmid DNA or 10× diluted gDNA ($\lambda$ ~ 0.6 for each), whereas for the 37 K array, the mixtures for duplicate reactions comprised either 1× diluted plasmid DNA or 2× diluted gDNA ($\lambda$ ~ 0.35 for each).

For the QX100 system, six different PPP concentrations (i.e., 300/300/100 nM, 300/300/200 nM, 600/600/100 nM, 600/600/200 nM, 900/900/100 nM, and 900/900/200 nM) were used with each combination of DNA template type and HCMV assay. The analyses were performed in duplicate with either 5× diluted plasmid DNA or 12.5× diluted gDNA ($\lambda$ ~ 0.07 for each). For each combination of PCR components, a single NTC was performed.

### Linearity, LOD, LOQ, and intraexperiment variability and repeatability on the qPCR and dPCR platforms

For the qPCR and both of the digital platforms, a single PPP concentration (i.e., 600/600/200 nM) in the *UL54* assay was used with Fast master mix (for qPCR and the 37 K array) or supermix for probes (for QX100) to quantify the dilution series of the gDNA. Fast master mix was selected for this, as it was more concentrated and therefore allowed higher volumes of sample input into the 37 K array inlets in comparison with the other three, less concentrated master mixes, whereas with the *UL54* assay, better data were obtained with the selected master mix in comparison with the *UL83* assay. To assess the qPCR and the QX100 system, 12 different dilutions of gDNA were used (starting from a 3.76× dilution), whereas the 37 K array for the Biomark system was tested with nine different gDNA concentrations (starting from a 1× dilution) (Tables S4, S5, S6). Additionally, for each platform, three to five NTCs were tested in each experiment. For the qPCR, the data from the dilution-series measurements from both days were combined and used to generate the standard curve, which allowed the calculation of the DNA concentrations for each individual measurement. The complete assessment was performed by one operator on two consecutive days, with five replicates of each dilution per day. The dilution series were freshly prepared on the day of analysis. To eliminate bias caused by possible unequal pipetting volumes during the sample mixing with the reaction mix, larger volumes of reaction mix and sample were mixed together, followed by the aliquoting of this mixture into five wells or inlets. For every dilution on each quantification platform, the nominal and measured mean *M*, *N* and $\lambda$ were calculated (Tables S4, S5, S6).

### Statistical analysis

The statistical analysis of the influence of the different PCR components on the DNA quantification was performed by single-factor analysis of variance and Student’s *t* tests (two tailed, two sample equal variance) in Microsoft Excel 2007. For the determination of outliers, Grubbs’s test (95 % confidence level) was used in Microsoft Excel 2007.

For assessment of linearity, LOD, LOQ and intraexperiment variability and repeatability, single-factor analysis of variance and Student’s *t* tests were used to determine the statistical significance of the bias between the nominal and measured concentrations. For each platform, intraexperiment linearity (five replicates from 1 day) and interexperiment linearity (ten replicates, five from each of 2 days) were analysed by means of Pearson’s correlation coefficients (R studio version 0.98.977). Outliers were not determined, to retain the complete variability of the data. For each platform, the LOD with a 95 % confidence interval was determined with Probit software, for which the interexperiment data were taken into account [26]. For each nominal DNA concentration, the coefficient of variation (CV) was calculated for either five or ten replicates, according to Eq. 5:

5 $$ CV=\frac{standard\kern0.5em  deviation}{average\kern0.5em  concentration} $$

The CVs for each platform were plotted against the nominal concentrations (in terms of either copies per total reaction volume or copies per effective reaction size) (R studio version 0.98.977). For each platform, the LOQ based on the interexperiment data was determined as the lowest nominal DNA concentration where the CV was still below 25 % [27]. The predicted Poisson error, expressed as the theoretical CV, was calculated for both of the dPCR platforms according to Eq. 6, as based on Poisson statistics [6]:

6 $$ CV(theoretical)=\sqrt{\left(\frac{1-{e}^{-\lambda }}{k{\lambda}^2{e}^{-\lambda }}\right)} $$

## Results

### Real-time PCR: high variability of Cq values obtained with different PCR components

**Fig. 2:**
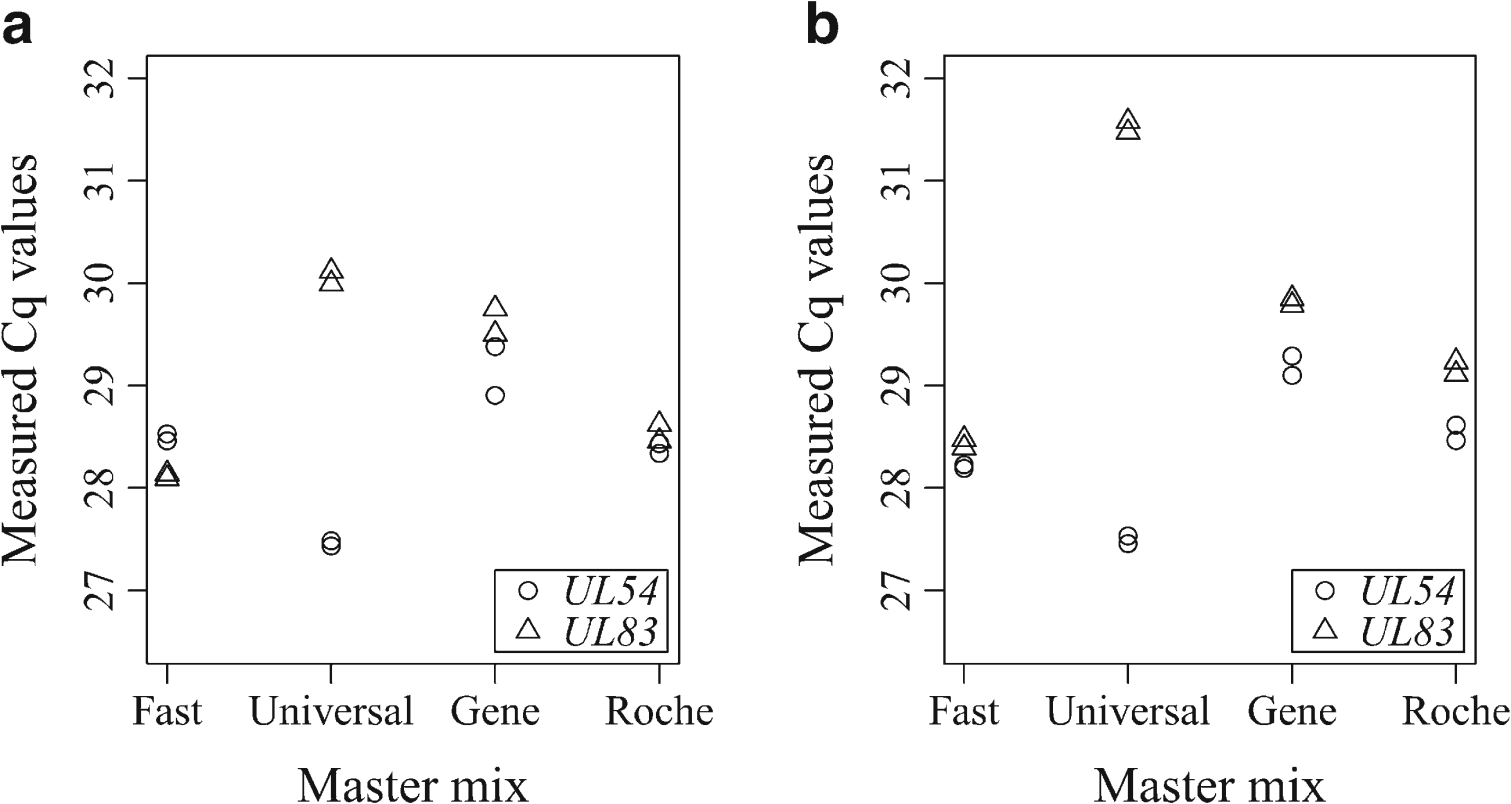
Effects of the different HCMV assays and master mixes on the Cq values for qPCR, with use of circular plasmid DNA (**a**) and genomic DNA (**b**). In a single run, each template type was amplified with each combination of HCMV assay and master mix, in duplicate

The initial qPCR study was performed with the two HCMV assays (i.e., *UL54* and *UL83*) and the four master mixes (i.e., Fast master mix, Universal master mix, Gene master mix and Roche master mix) on the plasmid DNA and gDNA, to select two master mixes for further testing for the Biomark platform (Fig. 2). The master mixes were selected according to the highest variability in Cq values between different HCMV assays and/or between different master mixes for the same HCMV assay. With each DNA template, Universal master mix demonstrated the largest differences in Cq values between the two HCMV assays. With the plasmid DNA, the fluorescence of the *UL54* assay reached the fluorescence threshold (Cq) 2.5 cycles before that of the *UL83* assay (Fig. 2a), whereas when gDNA was used, between the two HCMV assays there was a difference of 4 in the Cq values (Fig. 2b). With each DNA template, the other three master mixes gave only small differences in Cq values between the two HCMV assays (difference in Cq values, 0.1–0.7).

With the *UL83* assay, the largest differences in Cq values were observed with Fast master mix and Universal master mix (difference in Cq values, plasmid DNA 1.9, gDNA 3.1), whereas with the *UL54* assay, the largest differences were observed between Universal master mix and Gene master mix (difference in Cq values, plasmid DNA 1.7, gDNA 1.7) (Fig. 2). According to the data obtained, Fast master mix and Universal master mix were selected for further testing of the influence of different PCR components on DNA quantification for the Biomark system.

### Digital-PCR-based quantification shows resilience to the different PCR components

**Fig. 3:**
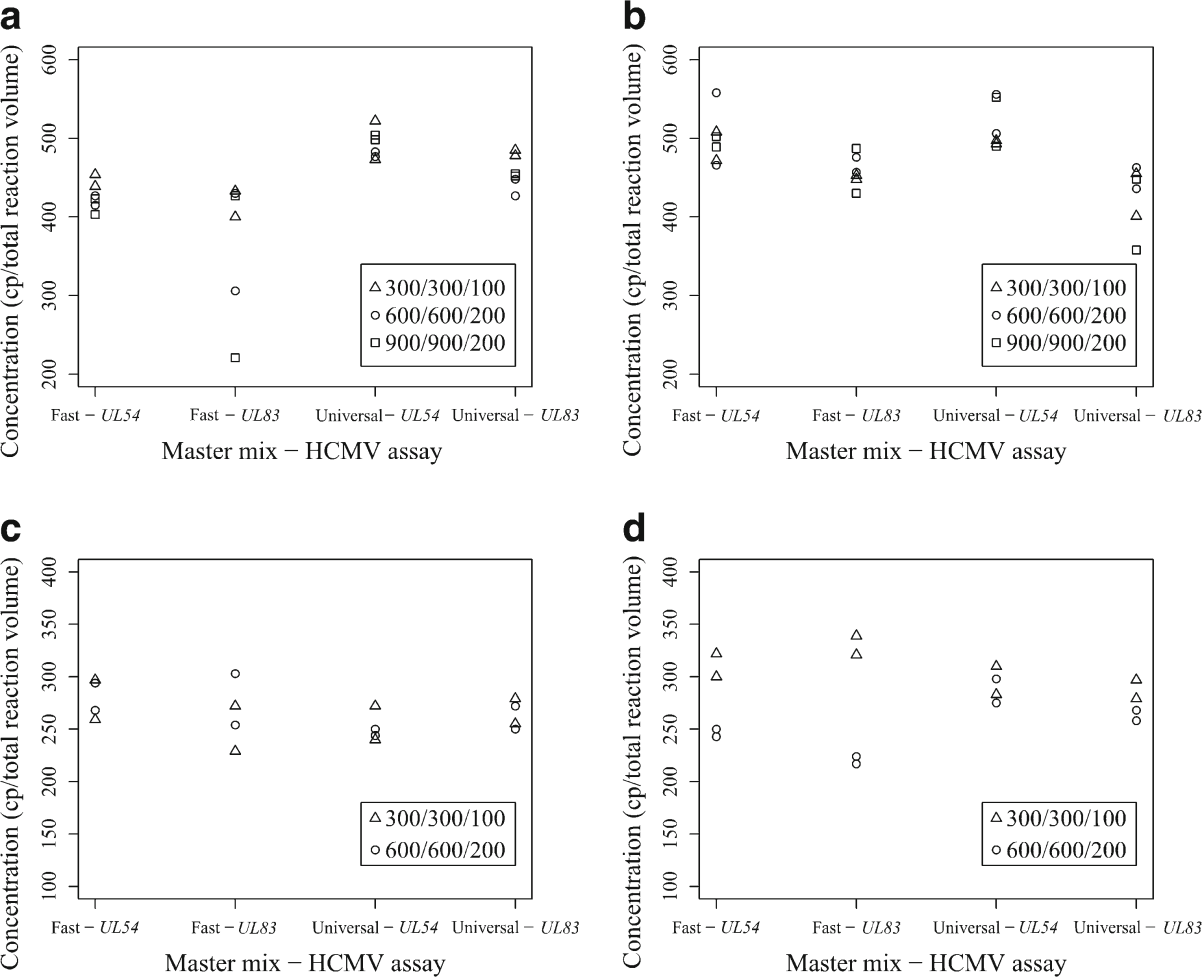
Influence of the different PCR components on the copy-number estimations for the Biomark system for the 12.765 array (**a**, **b**) and the 37 K array (**c**, **d**), with use of circular plasmid DNA (**a**, **c**) and genomic DNA (**b**, **d**). For each combination of HCMV assays and master mix, three (**a**, **b**) and two (**c**, **d**) different primer and probe concentrations were used (as indicated), with all experiments done in duplicate. *cp* copies

**Fig. 4:**
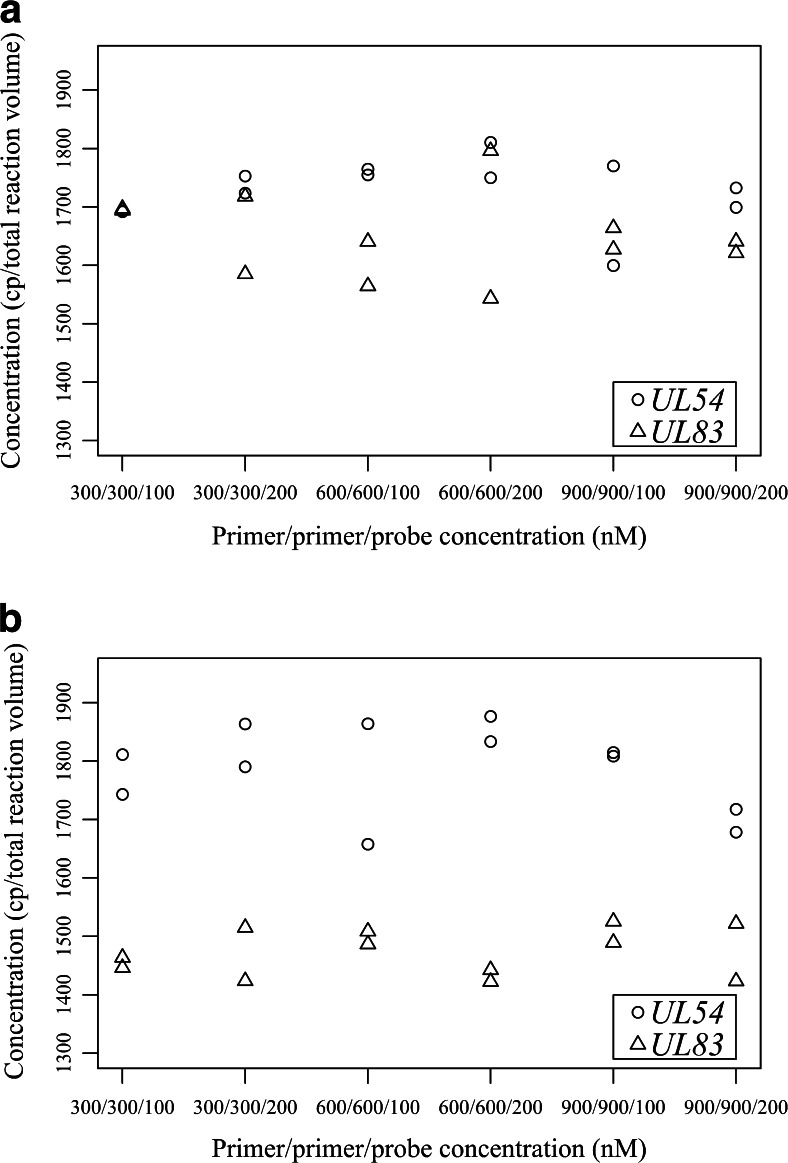
Influence of the different PCR components on the copy-number estimations for the QX100 system, with use of circular plasmid DNA (**a**) and genomic DNA (**b**). For each combination of HCMV assays and primer and probe concentrations, duplicates were used. *cp* copies

For both of the dPCR platforms, these assessments were performed with plasmid DNA and gDNA with the two HCMV assays with different PPP concentrations and both of the selected master mixes (Biomark) or supermix for probes (QX100). For each dPCR platform, there was little influence of the different PCR components on the DNA quantification (Figs. 3, 4).

On both arrays for the Biomark system, significant differences between the data were rarely observed (Fig. 3). With plasmid DNA for the 12.765 array (Biomark), the use of Universal master mix resulted in increased copy-number estimations (by 19 %) compared with Fast master mix, regardless of the HCMV assay or PPP concentrations (*p* < 0.01) (Fig. 3a). On the same array, differences were also observed with gDNA, as the *UL54* assay gave 15 % higher copy-number estimations than the *UL83* assay, regardless of master mix or PPP concentrations (*p* < 0.001) (Fig. 3b).

On the other hand, with the low PPP concentrations tested on the 37 K array in combination with the *UL83* assay, Fast master mix and gDNA, significant 37 % higher copy-number estimations were observed when compared with the estimations obtained when high PPP concentrations were tested (*p* < 0.001) (Fig. 3d). For each array, all of the other combinations of HCMV assay, PPP concentrations, master mixes and DNA templates did not show any statistically significant effects on the final copy-number estimations (*p* > 0.05). Additionally, on both arrays for the Biomark system, no outliers were detected. Furthermore, although there were up to twofold differences in the fluorescence intensities between the different PPP concentrations, and up to 2.5 Cq differences between the different HCMV assays and master mixes, there were no correlations with the previously mentioned significant differences in DNA copy-number estimations (Figs. S1, S2).

For the QX100 system with the *UL54* assay with gDNA, 20 % higher copy-number estimations were observed in comparison with the *UL83* assay, regardless of the PPP concentrations (*p* < 0.001) (Fig. 4b), whereas with plasmid DNA no such difference was found (Fig. 4a). Using the *UL54* assay with both of these DNA templates, we observed higher fluorescence intensities compared with those we observed when we used the *UL83* assay; however, both of these HCMV assays allowed simple manual threshold settings. With the different PPP concentrations, almost twofold differences in the fluorescence intensities of the droplets were observed (Fig. S3); however, there were no significant influences on the copy-number estimations (*p* > 0.05) (Fig. 4).

### Linearity, LOD, LOQ and intraexperiment variability and repeatability

For each platform, the linearity, LOD, LOQ and intraexperiment variability and repeatability were determined by means of serial dilutions of gDNA and one PPP concentration of the *UL54* assay, with either Fast master mix (qPCR and 37 K array) or supermix for probes (QX100). These measurements were performed over two consecutive days, with five replicates on each day.

### Linearity

**Fig. 5:**
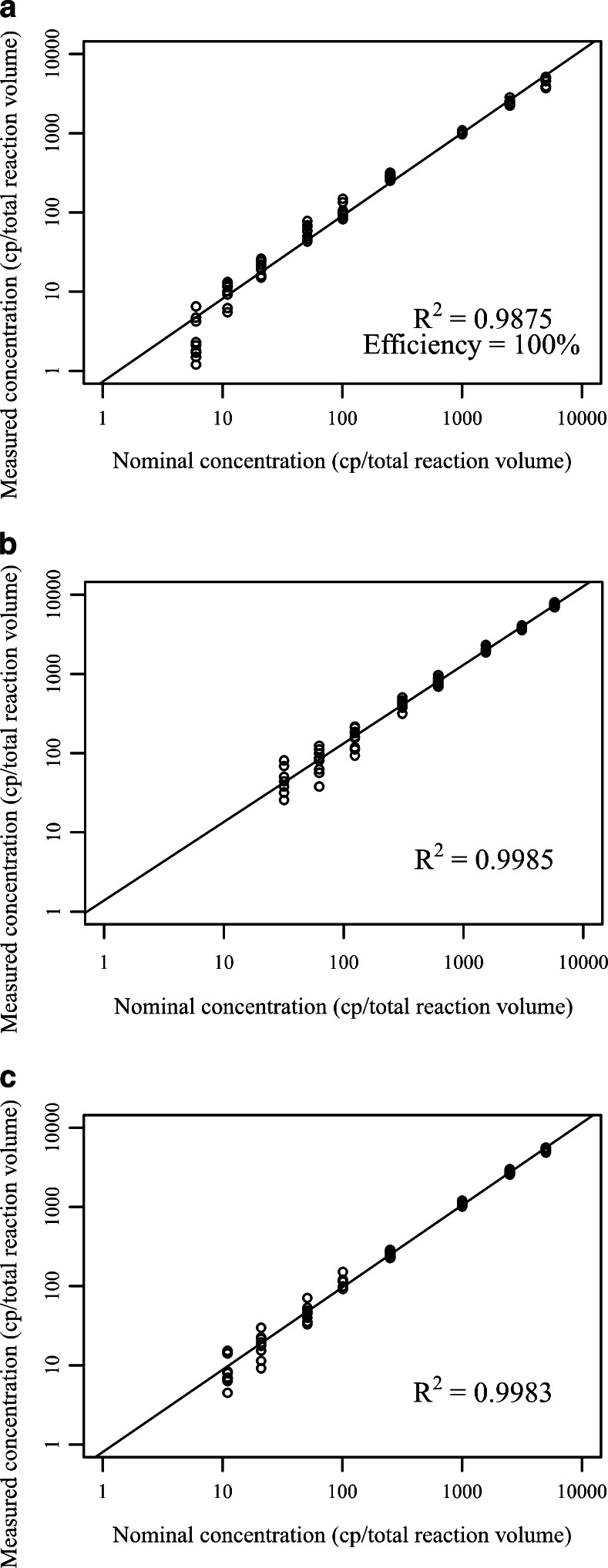
Interexperiment linearity for qPCR (**a**), the QX100 system (**b**), and the 37 K array for the Biomark system (**c**). DNA concentrations below the LODs were omitted from the plot. Each concentration of genomic DNA was measured in ten replicates, five from each of the 2 days. *cp* copies

Linear response above the LOD was observed, whereas for qPCR (measured linear dynamic range 5.5–5490 copies per total reaction volume), the Biomark system (measured linear dynamic range 33–6190 copies per total reaction volume) and the QX100 system (measured linear dynamic range 11–5490 copies per total reaction volume) (Fig. 5, Fig. S4), however, the upper dynamic range was not experimentally determined as high viral loads are not clinically relevant. On each dPCR platform, there was better correlation with nominal gDNA concentration (*R*^2^ > 0.998) in comparison with correlation on the qPCR platform (*R*^2^ > 0.987) (Fig. 5, Fig. S4).

### LOD, LOQ and intraexperiment variability and repeatability

**Fig. 6:**
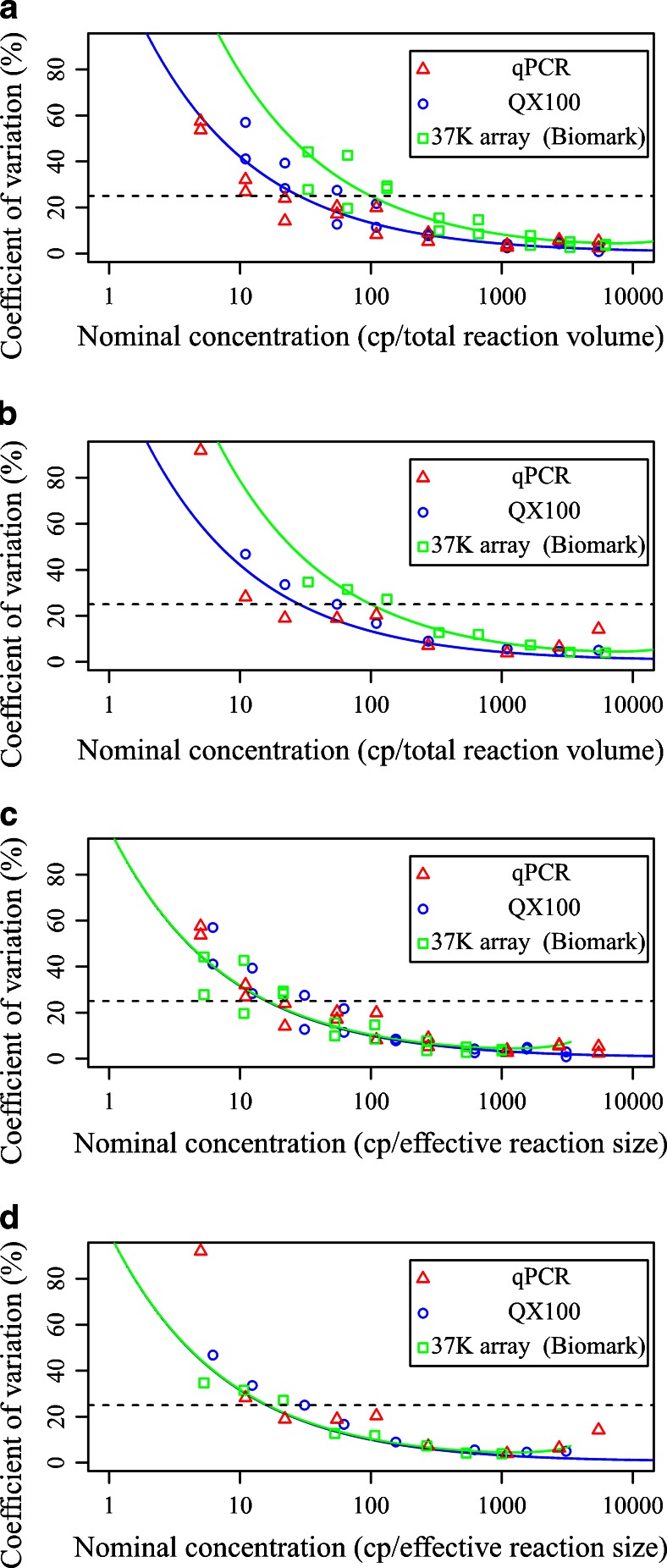
Intraexperiment variability (**a**, **c**) and repeatability (**b**, **d**) for all three of the platforms according to the total reaction volume (**a**, **b**) and the effective reaction size (**c**, **d**). The data are the coefficients of variation for five measurements on each day (**a**, **c**), or for ten combined measurements from 2 days (**b**, **d**). For each genomic DNA concentration, five replicates were measured on two consecutive days. *Blue line* theoretical coefficient of variation for the QX100 system (with 14,000 accepted droplets), *green line* theoretical coefficient of variation for the 37 K array for the Biomark system (with 770 chambers), *dashed line* criterion for the limit of quantification, set at a coefficient of variation of 25 %

On each platform, the data were assessed with two approaches, one based on the total reaction volume and the other based on the effective reaction size. When the total reaction volume was considered, the lowest LOD and lowest LOQ were obtained for qPCR, as the analysis indicated reliable detection and quantification of three and 11–22 copies, respectively (Table 1). For the QX100 system, the LOD was around six copies and the LOQ around 55 copies, whereas the 37 K array for the Biomark system showed the highest LOD (14 copies) and LOQ (140–190 copies) (Table 1). The qPCR platform and the QX100 system showed lower intraexperiment variability and higher repeatability in comparison with the 37 K array for the Biomark system (Fig. 6a, b). Furthermore, for qPCR, for concentrations below 55 copies, less variable data were obtained than for the QX100 system.

**Table 1:** Limit of detection (*LOD*) and limit of quantification (*LOQ*) for real-time PCR (*qPCR*) and both digital PCR (*dPCR*) platforms according to the reaction volume analysis. Only the interexperiment variability (repeatability) was taken into account. The LOD was calculated with Probit software. The LOQ was determined as the lowest concentration for which the coefficient of variation was below 25 %. The expected LODs and LOQs are in *parentheses*

Analysis method	Copy number according to analysis system					
	qPCR		QX100		Biomark	
	LOD	LOQ	LOD	LOQ	LOD	LOQ
Total reaction volume	2.8 (3)	11–22	5.7 (5.3)	55 (28)	14 (18)	140–210 (100)
Effective reaction size	2.8 (3)	11–22	3.2 (3)	30 (16)	2.4 (3)	14 (18)

In contrast, considering the effective reaction size on all three platforms, similar LODs were estimated (at around three copies). For concentrations above 55 copies, the repeatability for the dPCR platforms was equal to or better than that of qPCR. However, qPCR with concentrations between 10 and 55 copies showed the lowest LOQ, the lowest intraexperiment variability, and the highest repeatability (Fig. 6c, d).

On each platform, LODs equal to or lower than the theoretical LOD were observed (Table 1). On the other hand, on both of the dPCR platforms, the CV and LOQ from both the intraexperiment data and the interexperiment data were higher than the theoretical values (Table 1, Fig. 6).

#### Concordance between the platforms

On the qPCR platform and the QX100 system, inconsistent copy-number estimations were obtained throughout the dynamic range. On the qPCR platform, the copy-number estimations ranged from 14 % lower to 19 % higher than expected, whereas for the QX100 system, the estimations ranged from 12 % lower to 13 % higher than expected (Tables S4 and S6). For the Biomark system, very consistent copy-number estimations were obtained, which ranged from 19 to 26 % higher than expected (Table S5).

#### Cost-effectiveness and time-effectiveness for routine diagnosis of viruses and microorganisms

Clinical applicability of different platforms for routine diagnosis of HCMV and other pathogens also depends on their cost-effectiveness and time-effectiveness. Therefore, all three platforms were assessed in terms of relative final price per sample (consumables, labour fee and indirect costs), hands-on time and turnaround time to conduct the analysis for different numbers of samples (Table 2). Irrespective of the number of samples analysed, qPCR was the most cost-effective and time-effective. The QX100 system was more suitable for routine analysis than any array of the Biomark system, whereas 37 K array is more applicable for routine diagnosis than the 12.765 array. Additionally, in Slovenia, the price for the Biomark instrument is four to five times higher than that for qPCR instruments, whereas the cost of the QX100 instrument is up to two times higher than that of qPCR instruments.

**Table 2:** Comparison of cost-effectiveness and time-effectiveness between three platforms for analysis of clinical samples

Number of samples^a^	Items	qPCR	12.765 array (Biomark)	37 K array (Biomark)	QX100
1	Relative final price per sample (%)^b^	100	1339	614	137
	Hands on time (h)	1	1.2	1.4	1.25
	Total turnaround time (h)	2.8	3.2	4	3.5
5	Relative final price per sample (%)^b^	100	998	459	173
	Hands on time (h)	1.1	1.4	1.5	1.5
	Total turnaround time (h)	2.9	3.4	4.1	4
20	Relative final price per sample (%)^b^	100	2110	271	207
	Hands on time (h)	1.4	5.6	1.8	2
	Total turnaround time (h)	3.2	13.6	4.3	5
45	Relative final price per sample (%)^b^	100	2390	296	254
	Hands on time (h)	1.7	11.2	2.2	3
	Total turnaround time (h)	3.5	27.2	8.6	6.5

^a^For each analysis, every sample was estimated to be tested in duplicate, together with one negative template control and either a standard curve composed of five dilutions tested in duplicate (qPCR) or one positive control (both dPCR platforms)

^b^For the purpose of comparison, the final price per sample for qPCR analysis of 1, 5, 20, or 45 samples was taken as a reference with an assigned value of 100 %. Final prices were calculated on the basis of costs in Slovenia and include consumables, labour fees and indirect costs. Costs related to DNA extraction were excluded from the calculations

## Discussion

Digital PCR (dPCR) is becoming more and more recognized in molecular biology measurements for different applications, bringing new perspectives for absolute quantification of nucleic acids. Although some aspects of dPCR have already been investigated, in comparison with the present-day ‘gold standard’ of qPCR, there remains the need for further detailed evaluation and optimization of the analytical steps in dPCR procedures, to support accurate and reliable quantification of nucleic acids. In the present study, the assessment included qPCR, the QX100 system, and two different arrays for the Biomark system, with use of HCMV DNA, as accurate HCMV DNA quantification is of paramount importance for effective disease control [28, 29].

Different PCR components are known to have significant influences on the efficiency of qPCR, which can lead to different Cq values [30]. However, according to end-point measurements, dPCR has been reported to be more tolerant to differences in PCR efficiency than qPCR, which should thus result in little variation or no variations in copy-number estimations when different PCR components are used [8]. In the present study, two dPCR platforms were assessed for different combinations of HCMV assays and selected master mixes, which strongly influenced qPCR efficiency. Here, each dPCR platform showed low susceptibility to the influence of the different PCR components on the final copy-number estimations.

For the Biomark system, despite some minor effects of different HCMV assays, master mixes and PPP concentrations that altered the final copy-number estimations, these effects were never consistent for both DNA templates or both array types. Furthermore, these minor effects were probably not hampered by low PCR efficiency, which indicated that there are other causes for the observed differences in copy-number estimations. Little influence of different HCMV assays has already been reported [2, 6], which can be explained as a consequence of the different rates of molecular dropout for different HCMV assays [8, 31, 32]. Minor variability between different master mixes is also in agreement with previous reports, where differences of up to 50 % were observed [6, 32]. This can be explained by different influences of the master mixes, which can facilitate either molecular dropout or linkages between DNA templates, therefore increasing the proportion of partitions with more than one template, and consequently reducing the final copy-number estimation [8].

For the QX100 system, low susceptibility to different HCMV assays confirmed a previous observation [33]. However, the low influence of different HCMV assays might be caused by different degrees of molecular dropout, as already seen in our study on the 12.765 array for the Biomark system and in other reports [6]. In a study conducted on qPCR, the amplicon size was found to be inversely correlated with analytical sensitivity of qPCR [34]. Therefore, it could be speculated that the twofold difference in amplicon size between the two HCMV assays used in our study might be the major cause for the different rates of molecular dropout observed on each dPCR platform, since because of fragmented HCMV DNA, bigger amplicons are less likely to find an intact DNA fragment. High robustness of the QX100 system has already been confirmed, and this system can be efficiently used for multiplexing with varying PPP concentrations without hampering the quantification repeatability [35]. However, our finding is discrepant with that of a previous report, where there was a 66 % increase with the lower PPP concentrations compared with the higher PPP concentrations [20]. The main reason for the lower robustness in that previous report in comparison with the present findings might be the combination of small differences in the fluorescence intensities between the clusters of positive and negative droplets seen on the plots, and the high number of droplets with intermediate fluorescence (i.e., the ‘rain’), which was probably the result of either delayed amplification (i.e., the Monte Carlo effect) or differences in PCR efficiency of the particular assay [19]. It is reasonable to assume the combination of both of these factors might cause difficult discrimination between negative and positive droplets, particularly when low PPP concentrations are used, which would therefore hamper the decision on the final threshold setting and lead to overestimation or underestimation of the DNA copy numbers. Consequently, this might result in higher variability between different PPP concentrations. In contrast, the PCR efficiencies in the present study were near optimal for both of the HCMV assays, as the large increase in fluorescence intensities of the positive droplets and the small amount of rain resulted in straightforward setting of the threshold. Therefore, the degree of dPCR robustness might be closely related to general PCR efficiency of a particular HCMV assay, as efficient assays facilitate the distinction between the positive and the negative droplets, and are therefore more robust than assays with low efficiency [19].

When the assessment of linearity and variability was performed, between all three of these platforms, there was high agreement in the DNA copy-number estimations, although different PCR-based quantification techniques were compared. The highest agreement was noted between qPCR and the QX100 system. In theory, the qPCR estimation accuracy and agreement with estimations on dPCR usually depend on the choice of the method for quantification of the calibration material, and the commutability of the calibrant with the test samples when specific combinations of HCMV assay and master mix are used [29]. Consequently, compared with dPCR, qPCR can produce more than 50 % higher or lower DNA copy-number estimations [2, 7, 36]. In the present study, the agreement between qPCR and the QX100 system was high probably because of the use of gDNA that was initially quantified with the QX100 system, and that was used on the qPCR plaform as both the calibration material and the testing material, thus eliminating any reason for suboptimal commutability.

High agreement was also observed between the dPCR platforms; however, the Biomark system constantly gave higher copy-number estimations than the QX100 system. Good interplatform reproducibility of different dPCR platforms has already been documented in two previous assessmentes that compared the 37K array and 12.765 array for the Biomark system with the QX100 system [6, 18]. Although the Biomark system gave 8 % and 30 % higher estimations than the QX100 system, it is difficult to determine which platform, if any, provides the most accurate copy-number estimations. Therefore, either the overestimation of the DNA copy number for the Biomark system or the underestimation for the QX100 system, or the combination of both, should be taken into account. For the Biomark system, overestimation of DNA copy number could be due to either underestimation of chamber volumes on the 37 K array (based on Eq. 1) or the occurrence of single-stranded-DNA molecules during partitioning [8, 17, 32]. On the other hand, for the QX100 system, underestimation of the DNA copy number can be explained through several causes. As different master mixes were used on the dPCR platforms, the one used for the QX100 system might be associated with higher rates of molecular dropout and template linkages during partitioning. Additionally, improperly estimated partition volumes and their variability would also cause underestimation [8]. As only low mean partition occupancies were used in the present study for QX100, the impact of intracartridge partition variability should be negligible [8]. In contrast, overestimation of the mean droplet volume might have a bigger impact on the underestimation of the DNA copy numbers, as the estimated copy numbers are inversely related to the partition volume (Eq. 3). As the mean droplet volume ranges from 0.83 to 0.91 nL between laboratories and cartridges [3, 18, 37], different performances of droplet generators can be speculated.

Restricted sample volume input is considered the major factor for the superior analytical sensitivity of qPCR over dPCR [7, 9]. In the present study, qPCR was more sensitive than both of the dPCR platforms, which is in agreement with most other assessments [7, 9, 36]. However, equal LODs for qPCR and the QX100 system have also been reported [11]. Interestingly, the equal sample volumes used for qPCR and the QX100 system indicate that the restricted volume is probably not the only cause of the higher LOD for dPCR. Taking into account perfect assay specificity, the sample volume analysed must on average contain at least three copies to meet the criterion for the theoretical LOD (with 95 % probability) [1]. However, the volumetric difference between the initial sample input and the analysed sample volume (i.e., the effective reaction size) should also be taken into account when one is determining the theoretical LOD for the total reaction volume. Although in terms of the effective reaction size, on all three platforms the LOD was equal to the theoretical LOD, when the total reaction volume was considered, with both dPCR platforms higher LODs than with the qPCR platform were observed.

To our knowledge, this is the first comparison of the QX100 system and the 37 K array on the Biomark system in terms of linearity, analytical sensitivity and LOQ. Here, with respect to the total reaction volume when low concentrations were used (fewer than 1000 copies per total reaction volume), we indicate the dominant performance of the QX100 system in terms of LOD, LOQ and intraexperiment variability and repeatability. However, when the effective reaction size was considered, both platforms showed similar LOD, LOQ and intraexperiment variability and repeatability, although for the QX100 system, more than a 15-fold higher number of partitions was analysed. Furthermore, on both dPCR platforms, these data were also in agreement with the comparisons of theoretical CVs. Our findings strongly suggest that when the total reaction volume is considered, with low DNA concentrations, the variability of the dPCR platform is influenced not by the number of partitions analysed but by the ratio between the effective reaction size and the total reaction volume.

When the total reaction volume is considered, both dPCR platforms have been reported to show lower intraexperiment variability and higher repeatability throughout the entire dynamic range, in comparison with qPCR [2, 5, 9, 33]; however, such a trend was not observed in the present study. As the theoretical variability of dPCR is based on the Poisson distribution and can thus be mathematically predicted (Eq. 5), the qPCR variability is additionally dependent on the PCR efficiency and the instrument used, and consequently the variability is less predictable and is usually larger than the variability of dPCR [1, 23]. However, when the effective reaction size is considered, in the present study the variability of qPCR was not considerably higher that the theoretical variability of dPCR, which indicates low inhibition and only minor effects on amplification efficiency. As a result, when the total reaction volume is considered, the variability of qPCR was equal to, or even lower than, the theoretical variability of each of the dPCR platforms, probably due to the larger effective reaction size of qPCR. In addition, each dPCR platform showed higher variability than theoretically expected, which might also explain the slightly better performance of qPCR in comparison with both dPCR platforms, even when the effective reaction size was taken into account.

On both dPCR platforms, besides the matrix and HCMV assay effects, another source of higher variability than theoretically expected can be considered. On all three platforms, a certain sample volume is transferred from the tube into the wells or inlets, which acts as the first stochastic event. In addition, on both dPCR platforms, only certain parts of the total reaction volume are distributed from the wells or inlets into the chambers or droplets analysed, which is considered the second stochastic event. Therefore, when low DNA concentrations are used, it is reasonable to assume that unequal distribution of DNA templates during the additional stochastic events on the dPCR platforms might increase the variability. Despite a more than 20-fold difference in the analysed reaction volume between both dPCR platforms during the second stochastic event, equal variability was observed for both dPCR platforms when the effective reaction size was considered. This could indicate no or only a minor influence of the analysed reaction volume on the final variability, when a reaction volume of at least 0.64 μL is analysed. Nevertheless, additional statistical analysis should be done to confirm both hypotheses.

On both dPCR platforms, several approaches can be used to circumvent either small effective reaction sizes or restricted sample volumes. Although both of the arrays for the Biomark system have a constant effective reaction size because of fixed numbers of chambers on the array, the mean numbers of accepted droplets for the QX100 and QX200 systems vary, and will usually reach only 60–75 % of the initial 20,000 droplets [5, 11, 33]. Therefore, optimization of the pipetting techniques during the droplet transfer might increase the number of droplets accepted, which would theoretically result in an increased effective reaction size. Alternatively, the use of replicates might increase the effective reaction size analysed, and thus enhance the final sensitivity and repeatability [1].

For routine diagnosis of microorganisms and viruses, qPCR has been shown to be the most cost-effective and time-effective platform, which is in agreement with other studies [38, 39]. The minor differences in estimations of cost-effectiveness and time-effectiveness for qPCR and the QX100 system between the reports mentioned and our calculations are probably due to different prices for consumables, labour fees and indirect costs between countries, and different optimization of analysis protocols in terms of time.

## Conclusions

To the best of our knowledge, the present study represents the first assessment of the two most commonly used dPCR platforms, the QX100 system and both of the arrays for the Biomark system, performed in terms of the susceptibility to different PCR components when viral DNA is used. Additionally, we have reported the first evaluation of qPCR and both of the dPCR platforms in terms of linearity, LOD, LOQ and intraexperiment variability and repeatability, while considering both the total reaction volume and the effective reaction size. For the QX100 system and the Biomark system, there was only minor susceptibility to the different PCR components that hampered the qPCR assay efficiencies, thus confirming the high tolerance of dPCR. In addition, both of the dPCR platforms showed high interplatform agreement even in the low dynamic range of viral DNA concentrations. Minor discrepancies between different dPCR platforms might be further minimized if additional metrological studies to evaluate the partition volumes are performed. High resilience to inhibitors, assays with suboptimal efficiency and good reproducibility of different dPCR platforms might further indicate their potential suitability as higher-order reference measurement methods and as the methods for value assignment of reference materials [28]. Additionally, both dPCR platforms might be beneficial in the field of clinical diagnostics, especially when interlaboratory standardization of the qPCR-measured viral loads is limited by noncommutable standard materials [28]. Although the QX100 system, and especially the 37 K array for the Biomark system, reduced the applicability for quantification of low DNA concentrations because of the smaller effective reaction sizes compared with those in qPCR, the use of replicates and the optimization of pipetting techniques should help to increase their usefulness for such applications.

## Electronic supplementary material

ESM 1 (PDF 1295 kb)

## Abbreviations

Cq: Quantification cycle
CV: Coefficient of variation
dPCR: Digital PCR
Fast master mix: TaqMan® Fast Virus 1-Step Master Mix
gDNA: Genomic DNA
Gene master mix: TaqMan® Gene Expression Master Mix
HCMV: Human cytomegalovirus
LOD: Limit of detection
LOQ: Limit of quantification
MIQE: Minimum Information for Publication of Quantitative Real-Time PCR Experiments
NTC: Negative template control
PPP: Forward primer, reverse primer and probe
qPCR: Real-time PCR
Roche master mix: FastStart Universal Probe Master Mix
Universal master mix: TaqMan® Universal PCR Master Mix
