# Supplementary material for: Assessment of the real-time PCR and different digital PCR platforms for DNA quantification
Source: Anal Bioanal Chem. 2015 Oct 31;408:107–21. doi: 10.1007/s00216-015-9107-2 (PMC4706846; doi:10.1007/s00216-015-9107-2)

Analytical and Bioanalytical Chemistry

Electronic Supplementary Material

Assessment of the real-time PCR and different digital PCR platforms for DNA quantification

Jernej Pavšič, Jana Žel, Mojca Milavec

Table S1 MIQE checklist for authors, reviewers and editors

ITEM TO CHECK	IMPORTANCE	CHECKLIST
**EXPERIMENTAL DESIGN**		
Definition of experimental and control groups	**E**	Materials and methods
Number within each group	**E**	Materials and methods
Assay carried out by core lab or investigator's lab?	D	Investigator’s lab
Acknowledgement of authors' contributions	D	Author infotmation
**SAMPLE**		
Description	**E**	Materials and methods
Volume/mass of sample processed	D	Materials and methods
Microdissection or macrodissection	**E**	N/A
Processing procedure	**E**	Materials and methods
If frozen - how and how quickly?	**E**	N/A
If fixed - with what, how quickly?	**E**	N/A
Sample storage conditions and duration (especially for FFPE samples)	**E**	Materials and methods
**NUCLEIC ACID EXTRACTION**		
Procedure and/or instrumentation	**E**	Materials and methods
Name of kit and details of any modifications	**E**	Materials and methods
Source of additional reagents used	D	N/A
Details of DNase or RNAse treatment	**E**	No RNase/DNase treatment required
Contamination assessment (DNA or RNA)	**E**	N/A
Nucleic acid quantification	**E**	Materials and methods
Instrument and method	**E**	Materials and methods
Purity (A260/A280)	D	N/A
Yield	D	N/A
RNA integrity method/instrument	**E**	N/A
RIN/RQI or Cq of 3' and 5' transcripts	**E**	N/A
Electrophoresis traces	D	N/A
Inhibition testing (Cq dilutions, spike or other)	**E**	Fig. 5 and Fig. S4
**REVERSE TRANSCRIPTION**		
Complete reaction conditions	**E**	N/A
Amount of RNA and reaction volume	**E**	N/A
Priming oligonucleotide (if using GSP) and concentration	**E**	N/A
Reverse transcriptase and concentration	**E**	N/A
Temperature and time	**E**	N/A
Manufacturer of reagents and catalogue numbers	D	N/A
Cqs with and without RT	D*	N/A
Storage conditions of cDNA	D	N/A

Table S1 (cont.) MIQE checklist for authors, reviewers and editors

**qPCR TARGET INFORMATION**		
If multiplex, efficiency and LOD of each assay.	**E**	N/A
Sequence accession number	**E**	Table S3
Location of amplicon	D	N/A
Amplicon length	**E**	Table S3
In silico specificity screen (BLAST, etc)	**E**	NCBI PrimerBlast
Pseudogenes, retropseudogenes or other homologs?	D	N/A
Sequence alignment	D	N/A
Secondary structure analysis of amplicon	D	N/A
Location of each primer by exon or intron (if applicable)	**E**	N/A
What splice variants are targeted?	**E**	N/A
**qPCR OLIGONUCLEOTIDES**		
Primer sequences	**E**	Table S3
RTPrimerDB Identification Number	D	N/A
Probe sequences	D**	Table S3
Location and identity of any modifications	**E**	Table S3
Manufacturer of oligonucleotides	D	Table S3
Purification method	D	Table S3
**qPCR PROTOCOL**		
Complete reaction conditions	**E**	Materials and methods
Reaction volume and amount of cDNA/DNA	**E**	Materials and methods
Primer, (probe), Mg++ and dNTP concentrations	**E**	Materials and methods, N/A due to commercial kit
Polymerase identity and concentration	**E**	N/A due to commercial kit
Buffer/kit identity and manufacturer	**E**	N/A due to commercial kit
Exact chemical constitution of the buffer	D	N/A due to commercial kit
Additives (SYBR Green I, DMSO, etc.)	**E**	N/A due to commercial kit
Manufacturer of plates/tubes and catalog number	D	N/A
Complete thermocycling parameters	**E**	Materials and methods
Reaction setup (manual/robotic)	D	Manual setup
Manufacturer of qPCR instrument	**E**	Materials and methods

Table S1 (cont.) MIQE checklist for authors, reviewers and editors

**qPCR VALIDATION**		
Evidence of optimisation (from gradients)	D	N/A
Specificity (gel, sequence, melt, or digest)	**E**	Pure viral material was used
For SYBR Green I, Cq of the NTC	**E**	N/A
Standard curves with slope and y-intercept	**E**	Fig. 5, Fig. S4
PCR efficiency calculated from slope	**E**	Fig. 5, Fig. S4
Confidence interval for PCR efficiency or standard error	D	N/A
r2 of standard curve	**E**	Fig. 5, Fig. S4
Linear dynamic range	**E**	Fig. 5, Fig. S4
Cq variation at lower limit	**E**	Results
Confidence intervals throughout range	D	N/A
Evidence for limit of detection	**E**	Table 1
If multiplex, efficiency and LOD of each assay.	**E**	N/A
**DATA ANALYSIS**		
qPCR analysis program (source, version)	**E**	Materials and methods
Cq method determination	**E**	Manual determination of Cq values
Outlier identification and disposition	**E**	Materials and methods
Results of NTCs	**E**	No amplification was noticed in NTCs
Justification of number and choice of reference genes	**E**	N/A
Description of normalisation method	**E**	N/A
Number and concordance of biological replicates	D	N/A
Number and stage (RT or qPCR) of technical replicates	**E**	Materials and methods
Repeatability (intra-assay variation)	E	Fig. 6
Reproducibility (inter-assay variation, %CV)	D	Fig. 6
Power analysis	D	N/A
Statistical methods for result significance	**E**	Materials and methods
Software (source, version)	E	Materials and methods
Cq or raw data submission using RDML	**D**	N/A

MIQE checklist for authors, reviewers and editors. All essential information (E) must be submitted with the manuscript. Desirable information (D) should be submitted if available. If using primers obtained from RTPrimerDB, information on qPCR target, oligonucleotides, protocols and validation is available from that source.

*Assessing the absence of DNA using a no RT assay is essential when first extracting RNA. Once the sample has been validated as RDNA-free, inclusion of a no-RT control is desirable, but no longer essential

**Disclosure of the probe sequence is highly desirable and strongly encouraged. However, since not all commercial pre-designed assay vendors provide this information, it cannot be an essential requirement. Use of such assays is advised against

Table S2 dMIQE checklist for authors, reviewers and editors

ITEM TO CHECK	IMPORTANCE	CHECKLIST
**dPCR PROTOCOL**		
Complete reaction conditions	**E**	Materials and methods
Reaction volume and amount of cDNA/DNA	**E**	Materials and methods
Primer, (probe), Mg++ and dNTP concentrations	**E**	Materials and methods, some not available due to commercial kit
Polymerase identity and concentration	**E**	N/A due to commercial kit
Buffer/kit identity and manufacturer	**E**	Materials and methods
Exact chemical constitution of the buffer	D	N/A due to commercial kit
Additives (SYBR Green I, DMSO, etc.)	**E**	N/A due to commercial kit
Plates/tubes catalogue number and manufacturer	D	N/A
Complete thermocycling parameters	**E**	Materials and methods
Reaction setup (manual/robotic)	D	Manual setup
Gravimetric or volumetric dilutions (manual/robotic)	D	Volumetric dilutions (manual)
Total PCR volume prepared	D	Materials and methods
Partition number	**E**	Materials and methods
Individual partition volume	**E**	Materials and methods
Total volume of the partitions measured (effective reaction size)	**E**	Materials and methods
Partition volume variance/SD	D	N/A
Comprehensive details and appropriate use of controls	**E**	Materials and methods, Fig. S1, S2, S3
Manufacturer of dPCR instrument	**E**	Materials and methods
**dPCR VALIDATION**		
Optimisation data for the assay	D	N/A
Specificity (when measuring rare mutations, pathogen sequences etc)	**E**	In silico by blasting in NCBI
Limit of detection of calibration control	D	N/A
If multiplexing, comparison with singleplex assays	**E**	N/A

Table S2 (cont.) dMIQE checklist for authors, reviewers and editors

DATA ANALYSIS		
Mean copies per partition (λ or equivalent)	**E**	Table S5, S6
dPCR analysis program (source, version)	**E**	Materials and methods
Outlier identification and disposition	**E**	Materials and methods
Results of NTCs	**E**	Materials and methods, Fig. S3
Examples of positive(s) and negative experimental results as supplemental data	**E**	Materials and methods, Fig. S3
Where appropriate, justification of number and choice of reference genes	**E**	N/A
Where appropriate, description of normalization method	**E**	N/A
Number and concordance of biological replicates	D	N/A
Number and stage (RT or qPCR) of technical replicates	**E**	Materials and methods
Repeatability (intra-assay variation)	**E**	Fig. 6
Reproducibility (inter-assay/user/lab etc variation)	D	Fig. 6
Experimental variance or CI [d]	**E**	N/A
Statistical methods for analysis	**E**	Materials and methods
Data submission using RDML (Real-time PCR Data Markup Language)	D	N/A

dMIQE checklist for authors, reviewers and editors. All essential information (E) must be submitted with the manuscript. Desirable information (D) should be submitted if possible

[d] When single dPCR experiments are performed, the variation due to counting error alone should be calculated from the Binomial (or suitable equivalent) distribution.

Table S3 Information about amplicons, oligonucleotide primers and probes used with qPCR and dPCR

Assay name	Sequence accession number	Amplicon length (bp)	Type of oligonucleotide	Sequence (5'->3')	Manufacturer	Purification type
UL54	AY422361	72	Forward primer	GGCCGTTACTGTCTGCAGGA	Eurofins Scientific	Not provided by manufacturer
			Reverse primer	GGCCTCGTAGTGAAAATTAATGGT	Eurofins Scientific	Not provided by manufacturer
			Probe	CCGTATTGGTGCGCGATCTGTTCAA	Eurofins Scientific	HPLC
UL83	HM142972	159	Forward primer	GCAGCCACGGGATCGTACT	Eurofins Scientific	Not provided by manufacturer
			Reverse primer	GGCTACCTCACACGAGCATT	Eurofins Scientific	Not provided by manufacturer
			Probe	CGCGAGACCGTGGAACTGCG	Eurofins Scientific	HPLC

Table S4 List of nominal and measured gDNA concentrations on qPCR

Nominal DNA copy numbers [a]	Measured DNA copy numbers [b]	
M [c] ±SE	M [c] ± SE	Concordance (%) [d]
5,490 ±125	4,730 ±210	-14 ($p < 0.01$)
2,740 ±60	2,740 ±55	0
1,095 ±25	1,115 ±15	2
275 ±6	305 ±7	11 ($p < 0.01$)
110 ±2.5	110 ±7	3
55 ±1	65 ±4	19 ($p < 0.05$)
22 ±0.5	22 ±1.5	0
11 ±0.25	10 ±1	-6
5 ±0.1	2.3 ±0.7	-55 ($p < 0.001$)
2 ±0.05	ndf	/
1 ±0.02	ndf	/
0.1 ±0.002	ndf	/

[a] Based on initial estimations of gDNA on the QX100 system.

[b] Based on 10 replicates measured in two consecutive experiments.

[c] Number of DNA copies per total reaction volume with standard error (SE). This was equal to number of DNA copies per effective reaction size.

[d] The concordance between measured and nominal DNA concentration. Statistically insignificant bias ($p > 0.05$) have been omitted from the table.

Table S5 List of nominal and measured gDNA concentrations the 37K array on the Biomark system

Nominal DNA copy numbers [a]		Measured DNA copy numbers [b]			
M [c] ±SE	N [d] ±SE	M [c] ±SE	N [d] ±SE	λ [e]	Concordance (%) [f]
6,190 ±140	1,001 ±23	7,530 ±90	1,215 ±15	1.6	22 (p <0.001)
3,300 ±75	535 ±12	3,935 ±50	635 ±8.5	0.8	19 (p <0.001)
1,655 ±40	267 ±7	2049 ±45	330 ±7.5	0.4	24 (p <0.001)
660 ±15	107 ±2.5	790 ±30	130 ±5	0.2	19 (p <0.001)
330 ±8	54 ±1.2	418 ±17	68 ±3	0.08	26 (p <0.001)
132 ±3	21 ±0.5	165 ±13	26 ±2	0.03	24 (p <0.05)
66 ±1.5	11±0.2	85 ±8	13 ±1	0.02	26
33 ±0.8	5 ±0.1	50 ±5	8 ±0.7	0.01	45 (p <0.05)
6.6 ±0.1	1 ±0.02	ndf	ndf	ndf	/

[a] Based on initial estimations of gDNA on the QX100 system.

[b] Based on 10 replicates measured on two consecutive runs.

[c] Number of DNA copies per total reaction volume with standard error (SE).

[d] Number of DNA copies per effective reaction size with standard error (SE).

[e] Mean DNA copy number per partition.

[f] The concordance between measured and nominal DNA concentration. Statistically insignificant bias (p >0.05) have been omitted from the table.

Table S6 List of nominal and measured gDNA concentrations on QX100 system

Nominal DNA copy numbers [a]		Measured DNA copy numbers [b]			
M [c] ±SE	N [d] ±SE	M [c] ±SE	N [d] ±SE	λ [e]	Concordance (%) [f]
5.490 ±125	3.110 ±170	5.800 ±90	3.285 ±55	0.24	6 (p <0.05)
2.740 ±60	1.555 ±85	3.010 ±45	1.705 ±25	0.12	10 (p <0.001)
1.095 ±25	620 ±35	1.185 ±22	700 ±13	0.05	13 (p <0.05)
275 ±6	155 ±8	275 ±7	155 ±5	0.01	0
110 ±2.5	62 ±3.5	120 ±6	70 ±3	0.005	9
55 ±1	31 ±1.7	48 ±4	27 ±2	0.002	-12
22 ±0.5	12 ±0.7	19 ±2	11 ±1.2	0.001	-12
11 ±0.25	6 ±0.4	10 ±1.5	6 ±0.8	0.0004	-11
5 ±0.1	3 ±0.2	6 ±1	3 ±0.5	0.0002	8
2 ±0.05	1 ±0.06	ndf	ndf	0.00010	/
1 ±0.02	0.6 ±0.03	ndf	ndf	0.00005	/
0.1 ±0.002	0.06 ±0.03	ndf	ndf	0.000005	/

[a] Based on initial estimations of gDNA on the QX100 system.

[b] Based on 10 replicates measured in two consecutive runs.

[c] Number of DNA copies per total reaction volume with standard error (SE).

[d] Number of DNA copies per effective reaction size with standard error (SE).

[e] Mean DNA copy number per partition.

[f] The concordance between measured and nominal DNA concentration. Statistically insignificant bias (p >0.05) have been omitted from the table.

Fig. S1 Detailed dPCR data from assessment of influence of different PCR components on DNA quantification using the 12.765 array for the Biomark system

qPCR charts obtained either on 12.765 array for the Biomark system using undiluted plasmid DNA **(A)** or two-fold diluted genomic DNA **(B)**. Above each group of three qPCR charts, the array type, the template type, the master mix type and the HCMV assay type are depicted. Numbers above each chart depict different nM concentrations of forward primer, reverse primer and probe. The fluorescence threshold was determined manually for each combination of reaction components, while the quality threshold (0.2) and the accepted Cq range (15-45 Cq) were equal for all samples.

A)

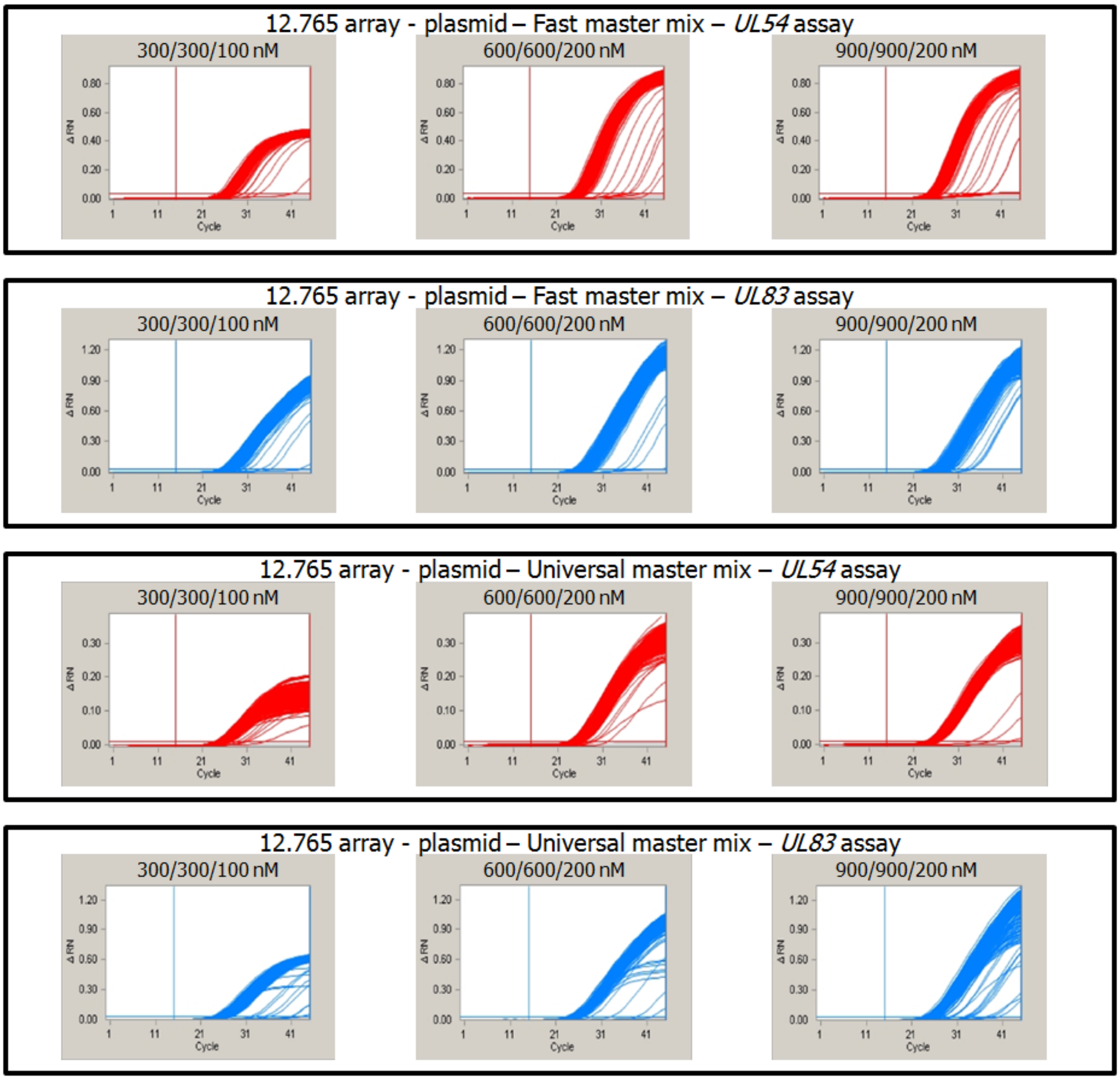

B)

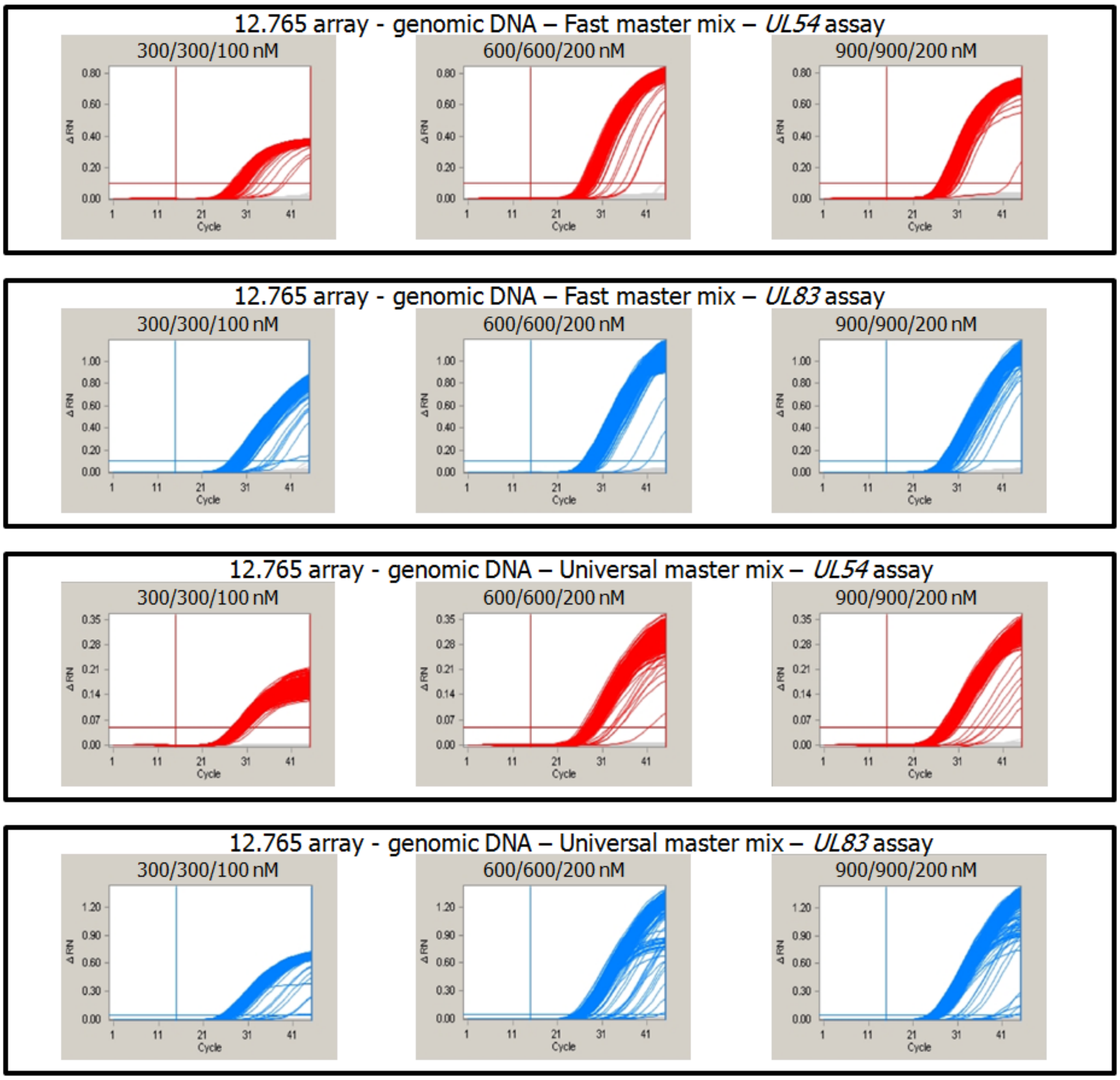
12.765 array - genomic DNA – Fast master mix – UL54 assay
300/300/100 nM
600/600/200 nM
900/900/200 nM
12.765 array - genomic DNA – Fast master mix – UL83 assay
300/300/100 nM
600/600/200 nM
900/900/200 nM
12.765 array - genomic DNA – Universal master mix – UL54 assay
300/300/100 nM
600/600/200 nM
900/900/200 nM
12.765 array - genomic DNA – Universal master mix – UL83 assay
300/300/100 nM
600/600/200 nM
900/900/200 nM
Δ RN
Cycle

Fig. S2 Detailed dPCR data from assessment of influence of different PCR components on DNA quantification using the 37K array for the Biomark system

qPCR charts obtained on the 37K array for the Biomark system using either 1× diluted plasmid DNA **(A)** or 2× diluted genomic DNA **(B)**. Above each pair of qPCR charts, the array type, the template type, the master mix type and the HCMV assay type are described. Numbers above each chart depict different nM concentrations of forward primer, reverse primer and probe. The fluorescence threshold was determined manually for each combination of reaction components, while the quality threshold (0.2) and the accepted Cq range (15-45 Cq) were equal for all samples.

A)

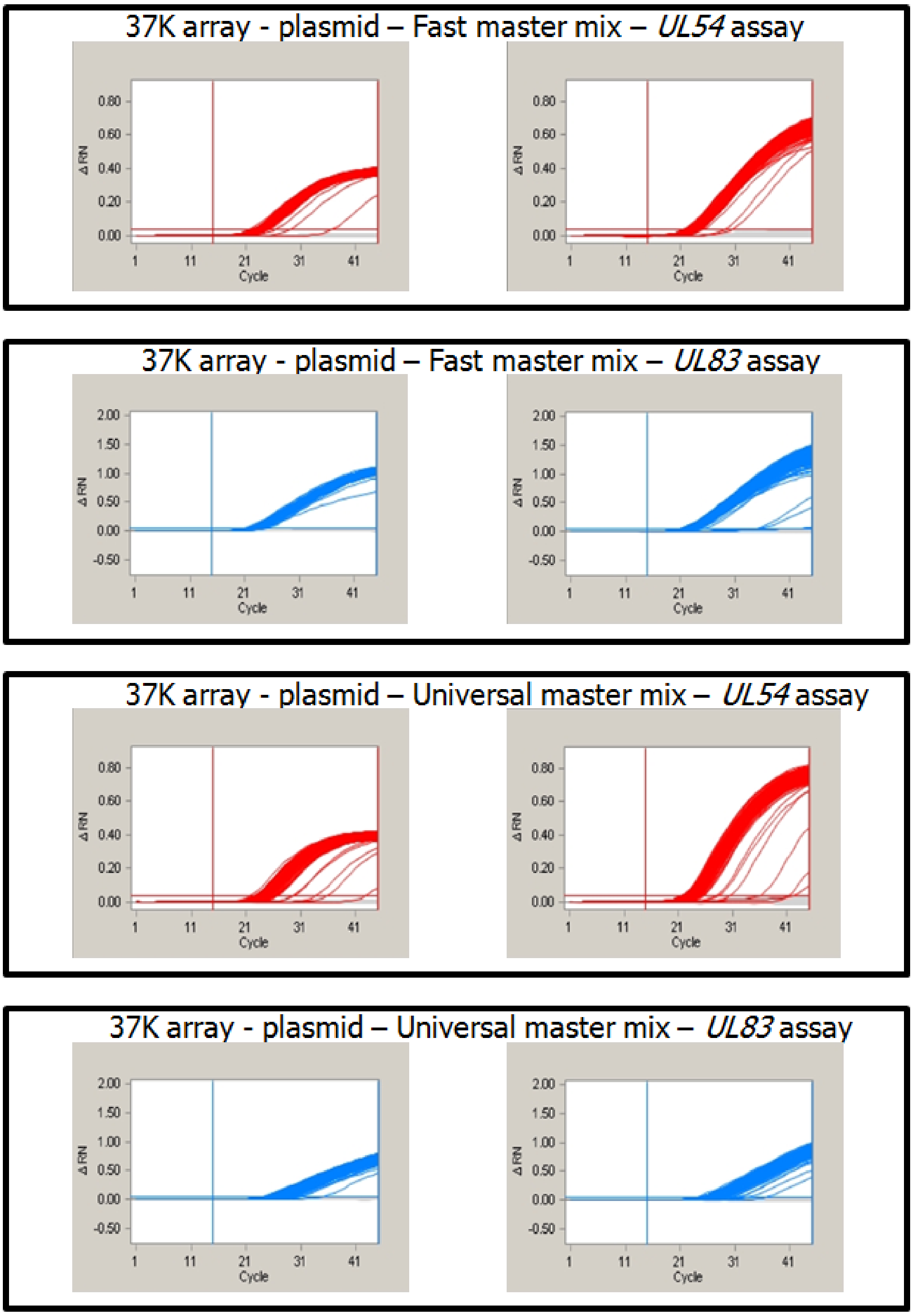

B)

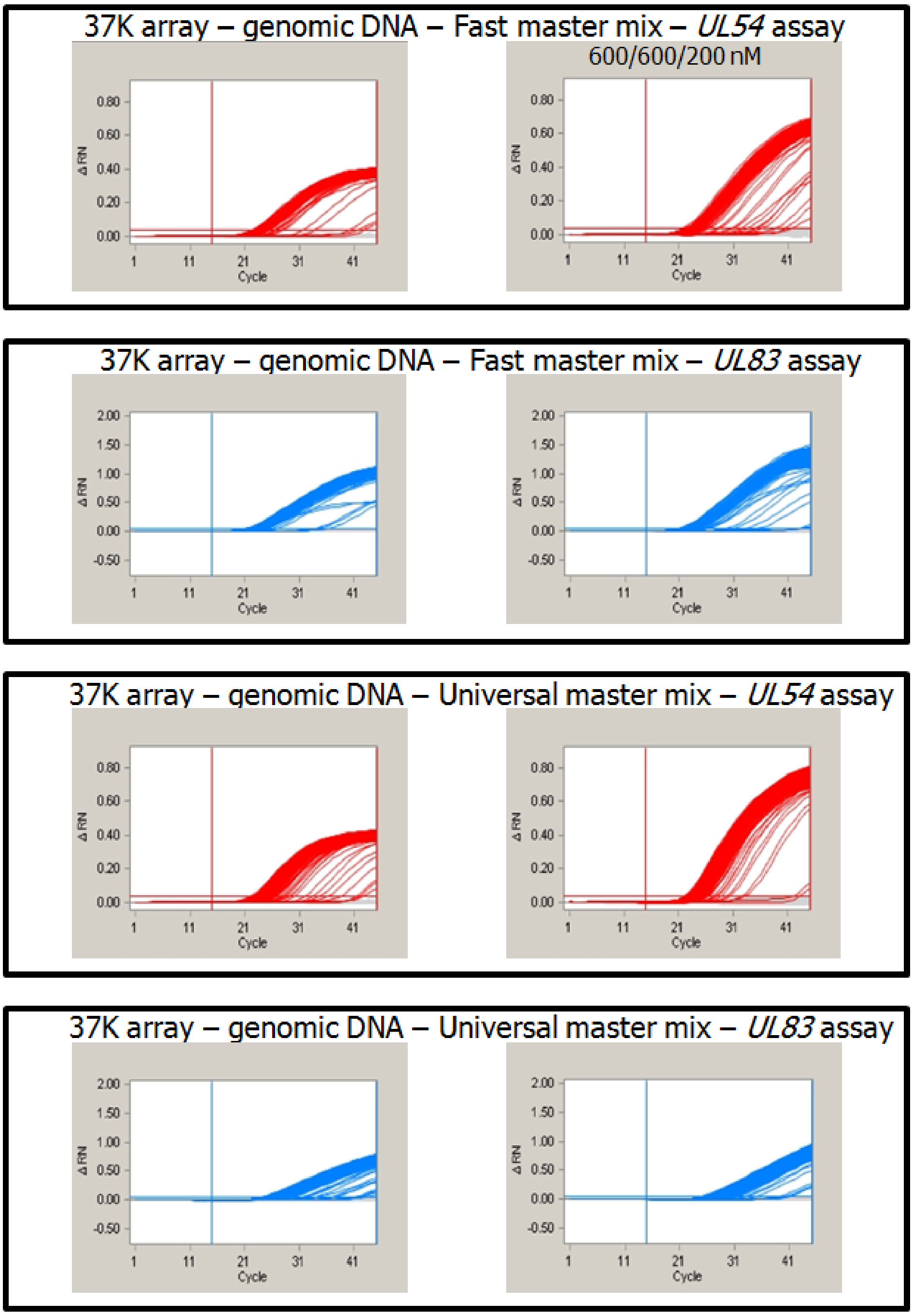
37K array – genomic DNA – Fast master mix – *UL54* assay
600/600/200 nM
Δ RN
Cycle
37K array – genomic DNA – Fast master mix – *UL83* assay
Δ RN
Cycle
37K array – genomic DNA – Universal master mix – *UL54* assay
Δ RN
Cycle
37K array – genomic DNA – Universal master mix – *UL83* assay
Δ RN
Cycle

Fig. S3 Detailed dPCR data from assessment of influence of different PCR components on DNA quantification using the QX100 system

Fluorescence intensity of droplets obtained on the QX100 system using *UL54* assay **(A)** or *UL83* assay **(B)**. Each dot represents fluorescence intensity for particular droplet. Numbers above each column depict different nM concentrations of forward primer, reverse primer and probe. Except of NTCs, each assay was tested with two-fold diluted gDNA. The threshold was set manually.

A)

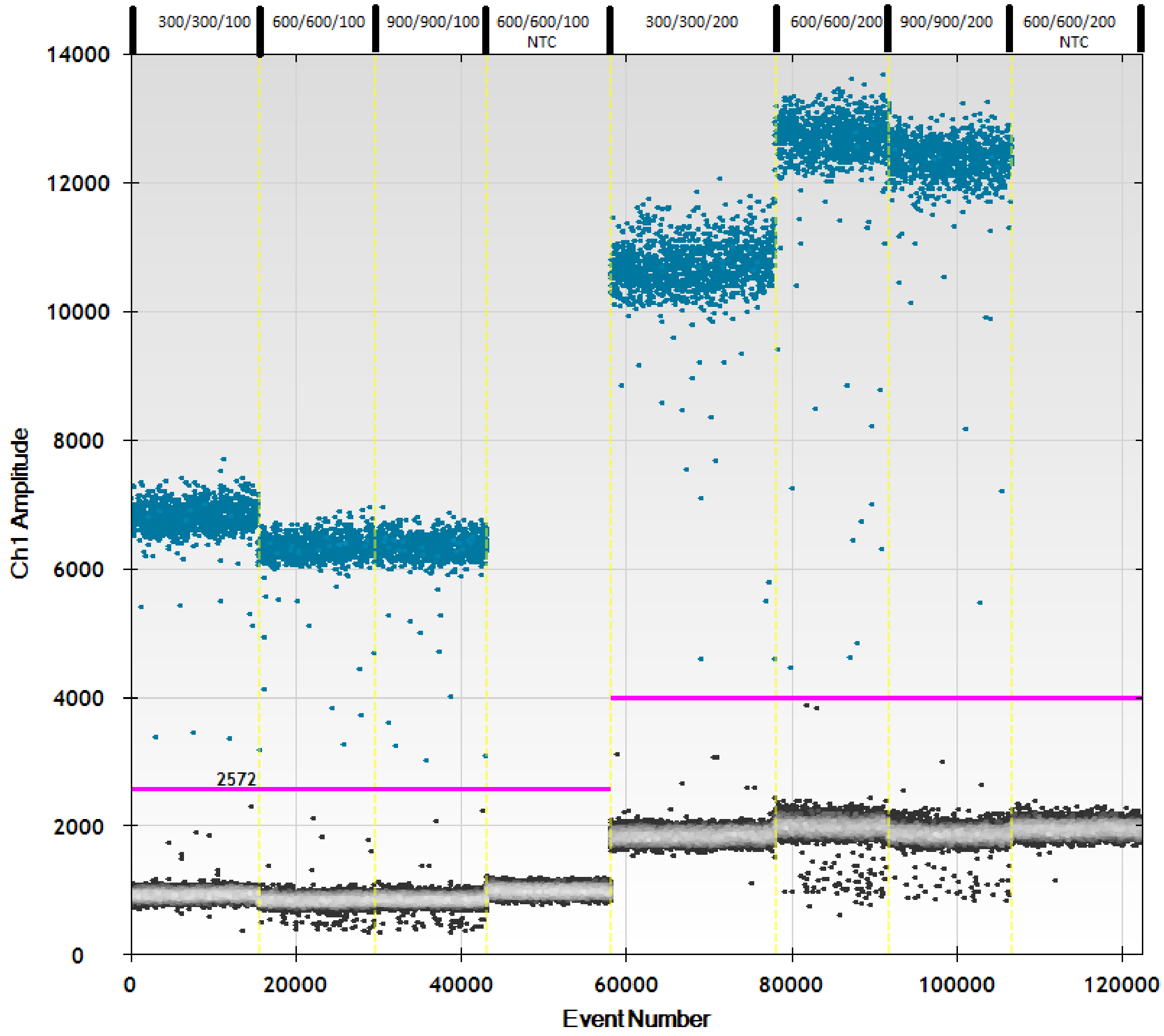

B)

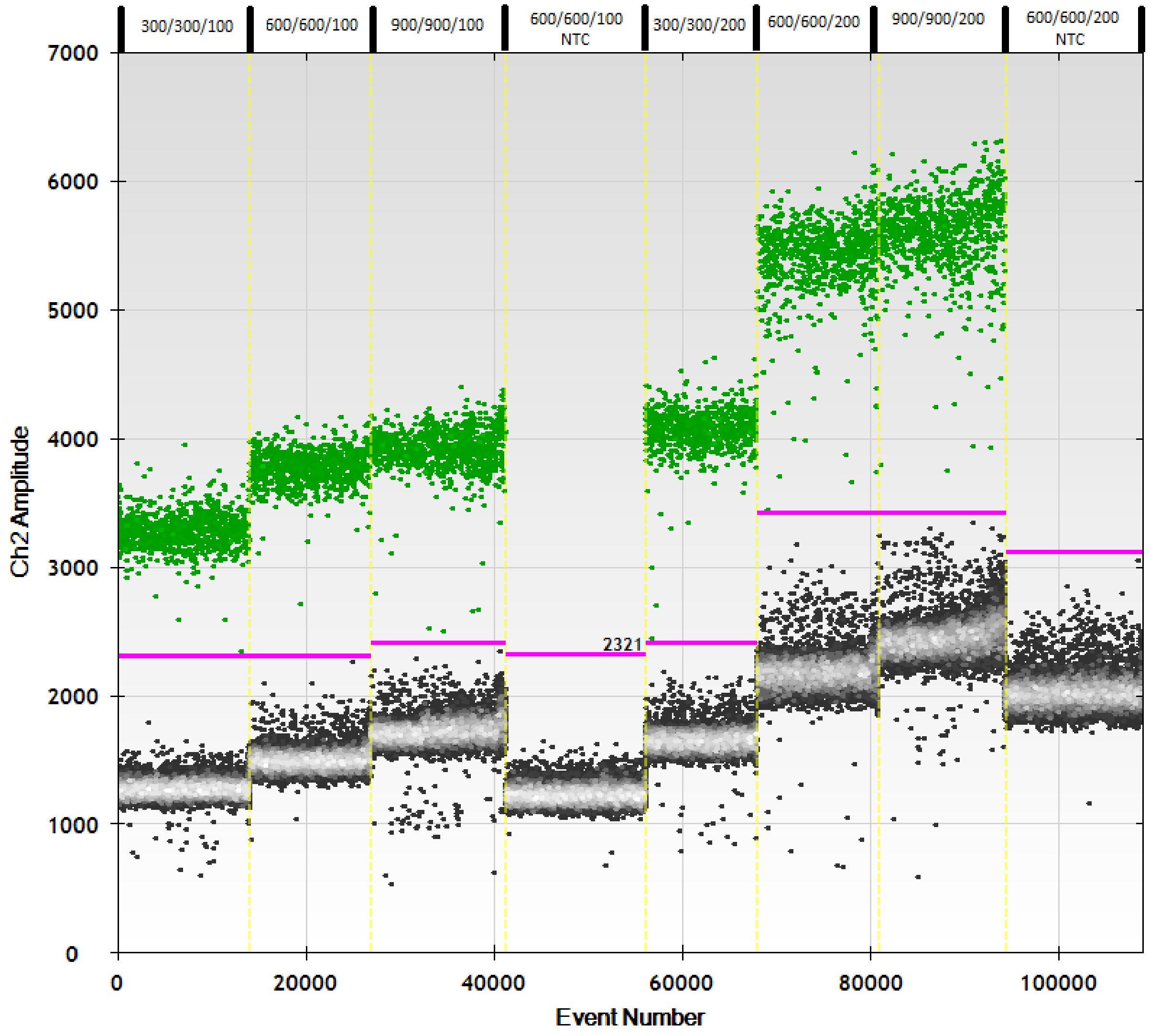

300/300/100
600/600/100
900/900/100
600/600/100 NTC
300/300/200
600/600/200
900/900/200
600/600/200 NTC
7000
6000
5000
4000
3000
2000
1000
0
Ch2 Amplitude
2321
0
20000
40000
60000
80000
100000
Event Number

Fig. S4 Intra-experiment linearity

Intra-experiment linearity of qPCR **(A)**, the 37K array for the Biomark system **(B)** and the QX100 system **(C)**. DNA concentrations below LOD were omitted from the plots. Each concentration of genomic DNA was measured in five replicates on each of two days. Each day is denoted by different colour.

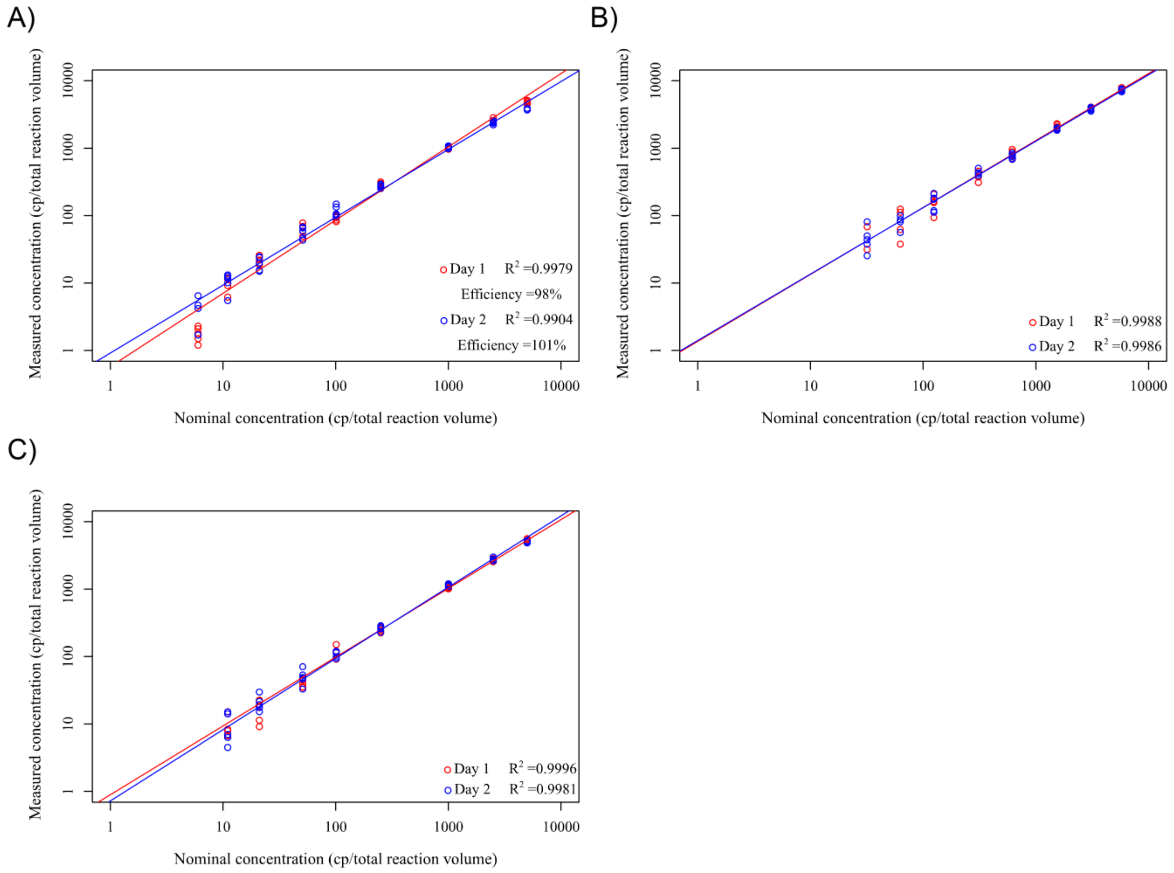

Supplement: Supplementary file 1 — (PDF 1295 kb) [file 216_2015_9107_MOESM1_ESM.pdf]
